# Supplementary figures and images for: A Population Genetic Signal of Polygenic Adaptation
Source: PLoS Genet. 2014 Aug 7;10(8):e1004412. doi: 10.1371/journal.pgen.1004412 (PMC4125079; doi:10.1371/journal.pgen.1004412)

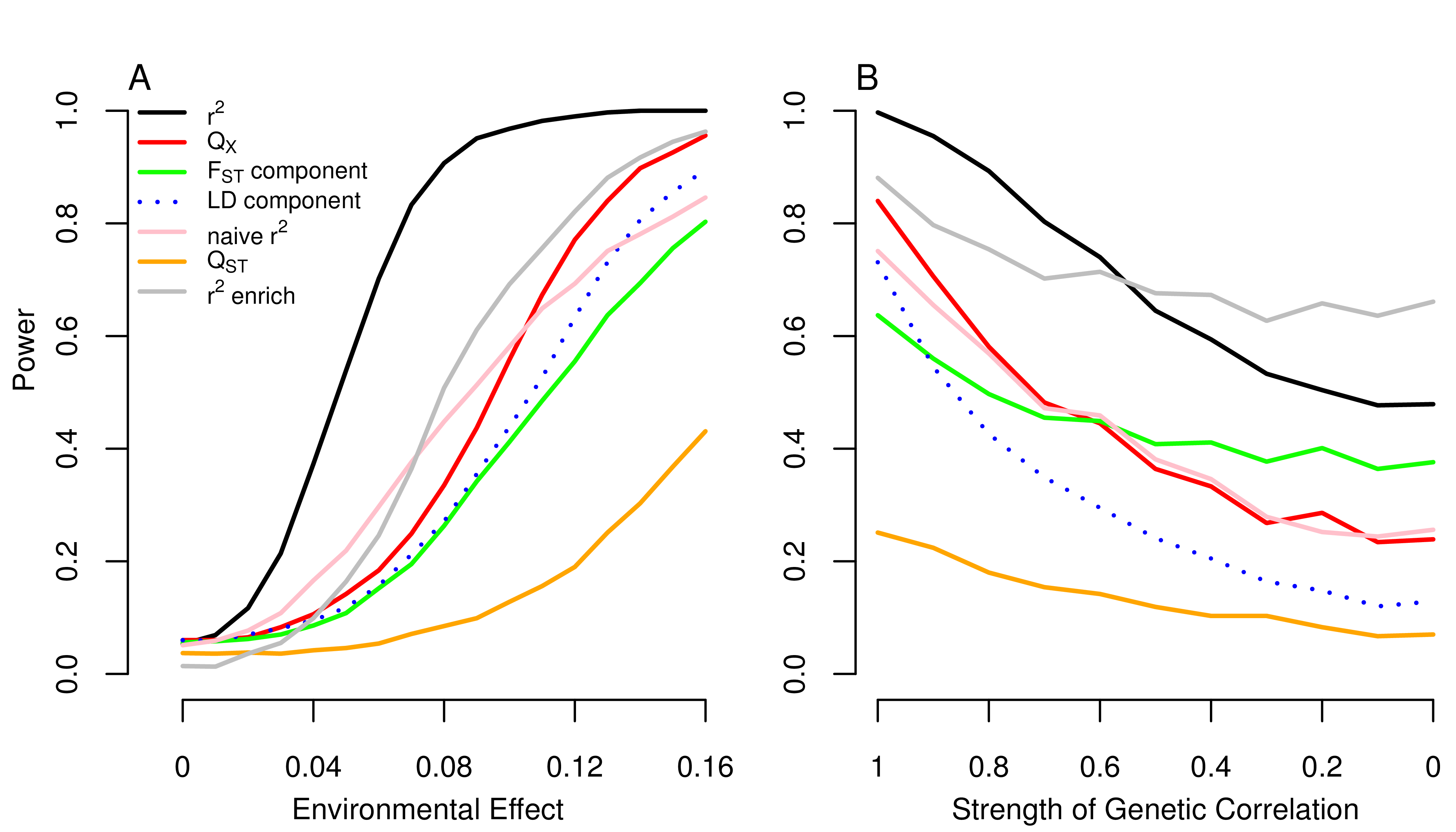

Supplement: Figure S1 — Power of tests described in the main text to detect a signal of selection on the mapped genetic basis of skin pigmentation [67] as an increasing function of the strength of selection (A), and a decreasing function of the genetic correlation between skin pigmentation and the selected trait with the effect of selection held constant at (B). (TIFF) [file pgen.1004412.s001.tiff]

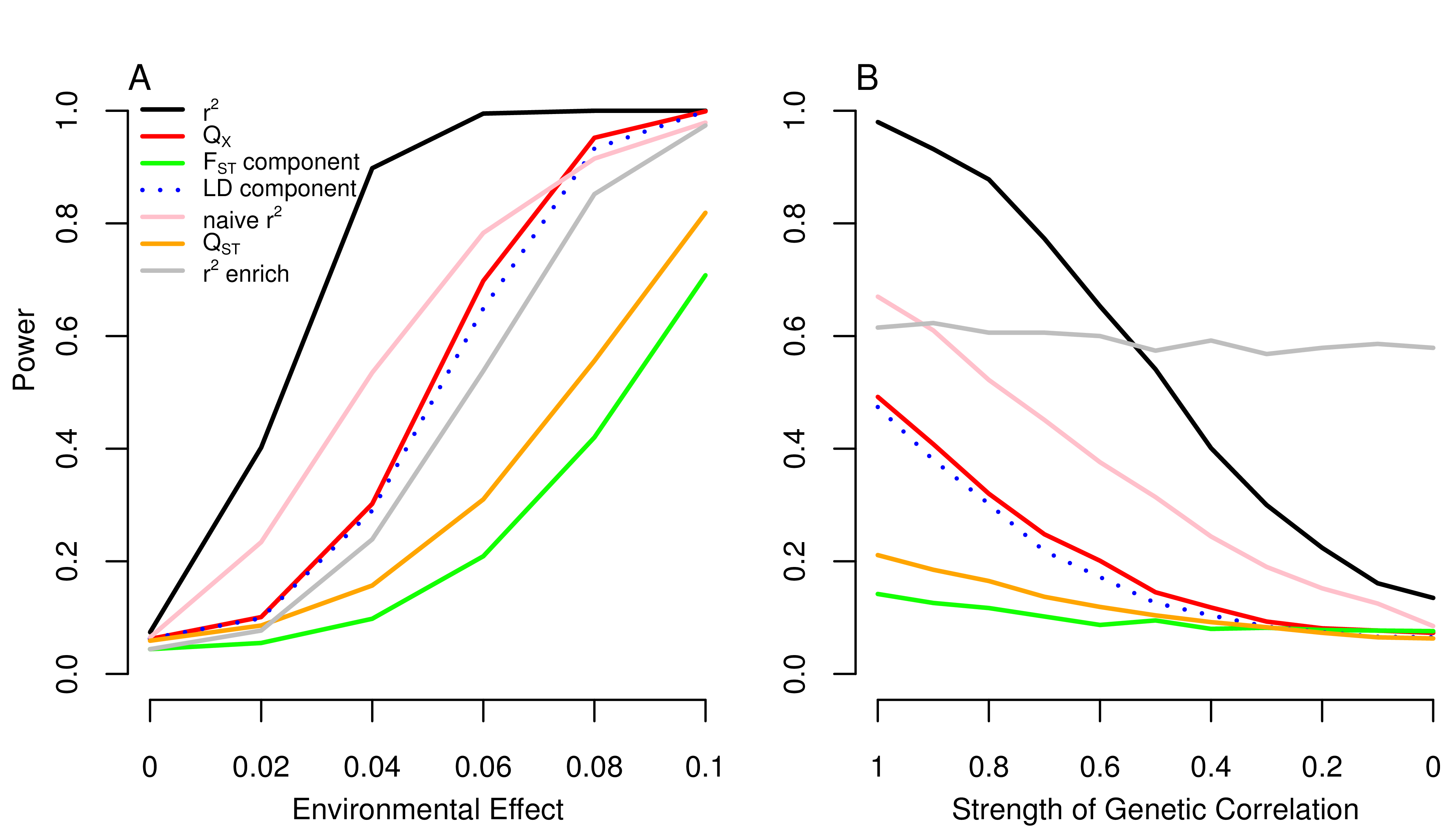

Supplement: Figure S2 — Power of tests described in the main text to detect a signal of selection on the mapped genetic basis of BMI [74] as an increasing function of the strength of selection (A), and a decreasing function of the genetic correlation between BMI and the selected trait with the effect of selection held constant at (B). (TIFF) [file pgen.1004412.s002.tiff]

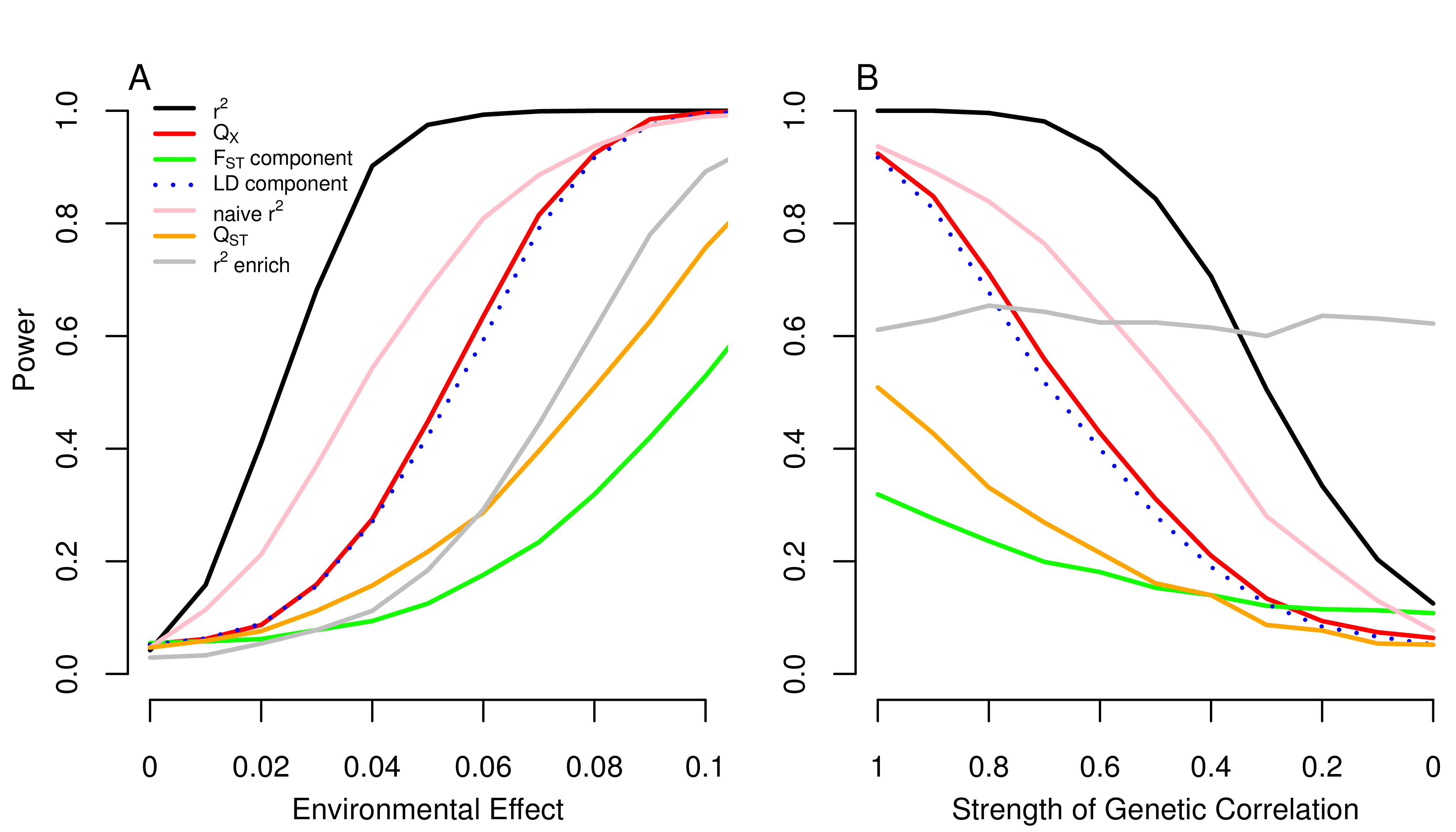

Supplement: Figure S3 — Power of tests described in the main text to detect a signal of selection on the mapped genetic basis of T2D [75] as an increasing function of the strength of selection (A), and a decreasing function of the genetic correlation between height and the selected trait with the effect of selection held constant at (B). (TIFF) [file pgen.1004412.s003.tiff]

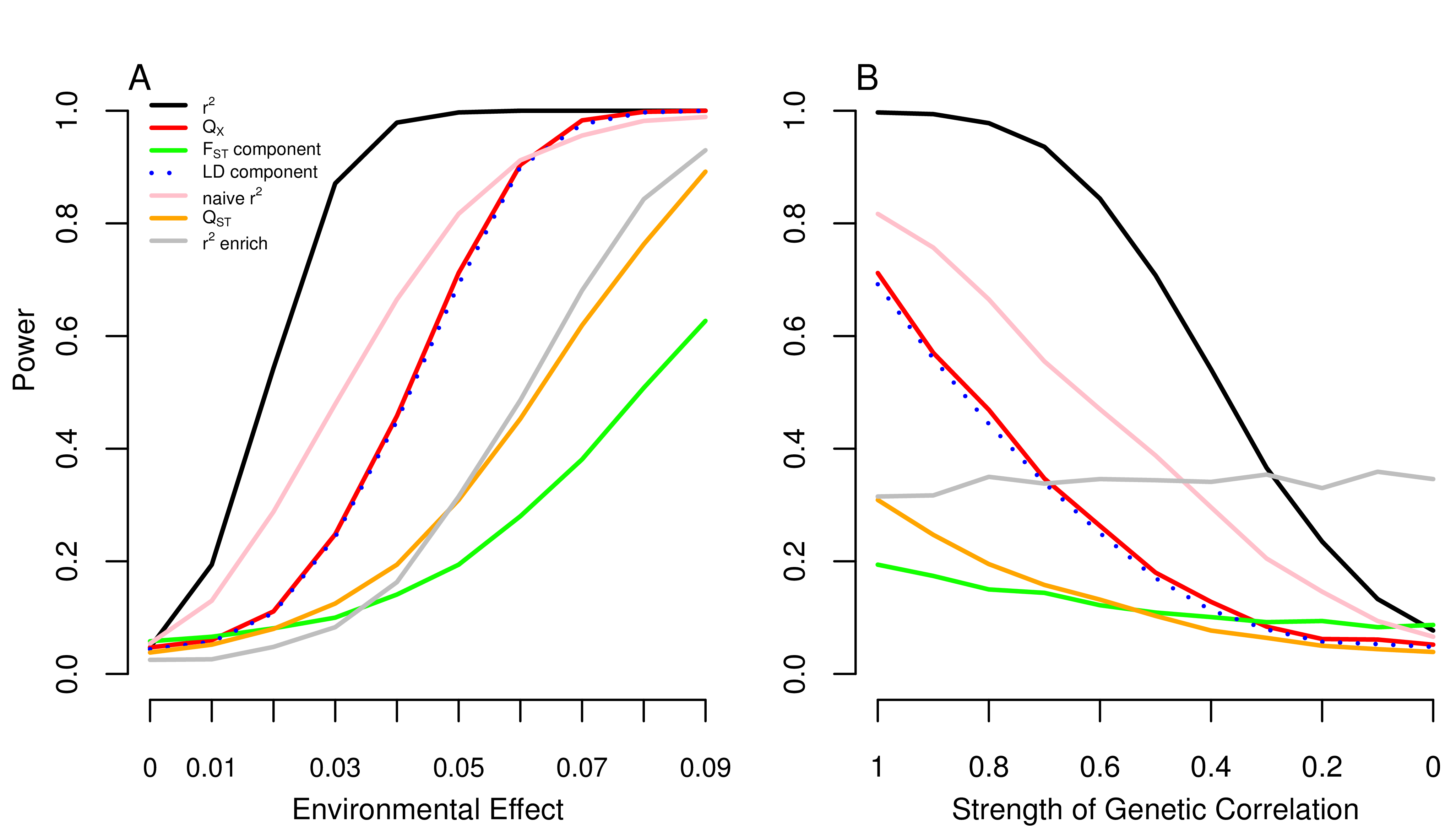

Supplement: Figure S5 — Power of tests described in the main text to detect a signal of selection on the mapped genetic basis of UC [26] as an increasing function of the strength of selection (A), and a decreasing function of the genetic correlation between UC and the selected trait with the effect of selection held constant at (B). (TIFF) [file pgen.1004412.s005.tiff]

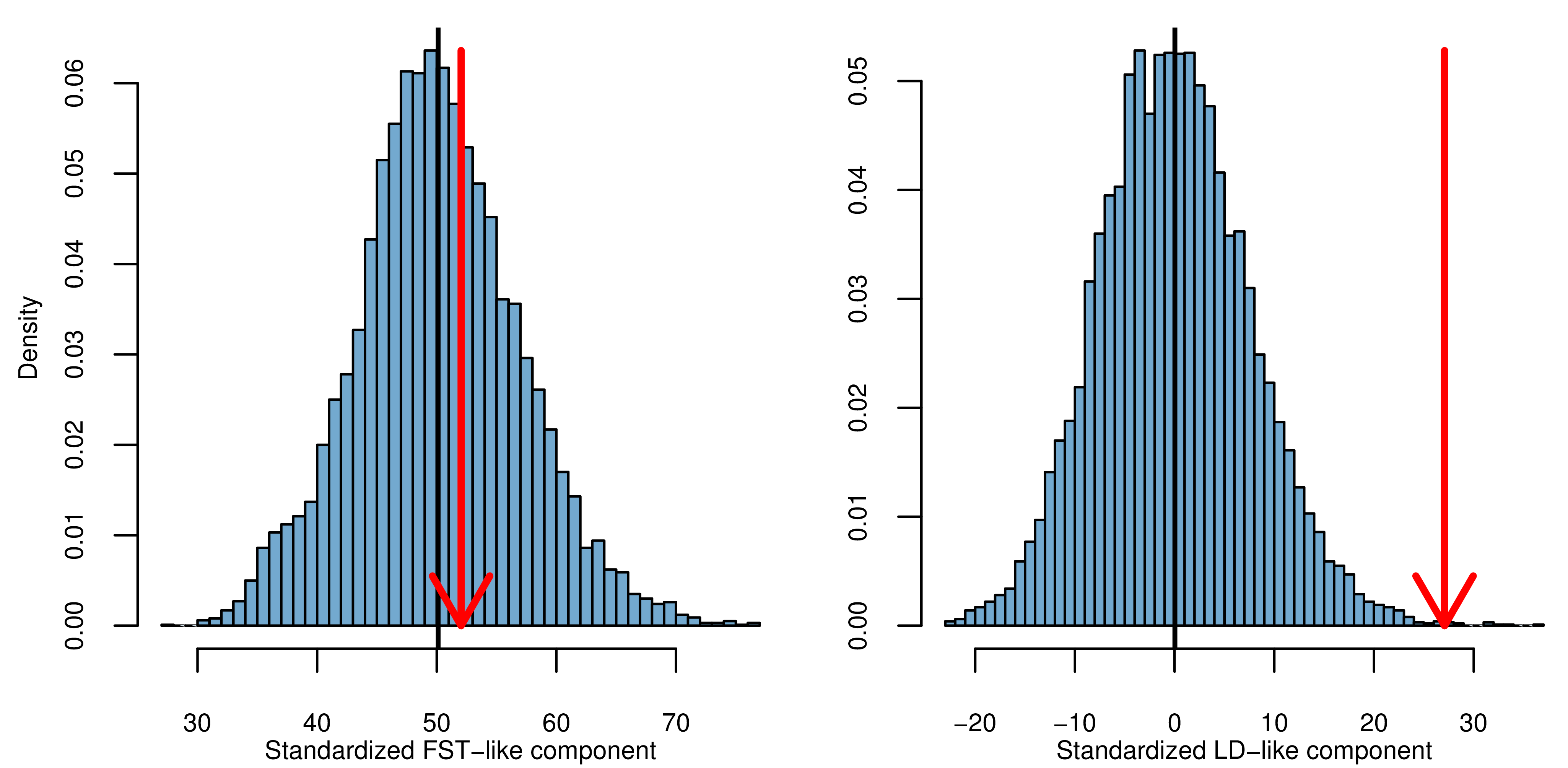

Supplement: Figure S6 — The two components of for the skin pigmentation dataset, as described by the left and right terms in (14). The null distribution of each component is shows as a histogram. The expected value is shown as a black bar, and the observed value as a red arrow. (TIFF) [file pgen.1004412.s006.tiff]

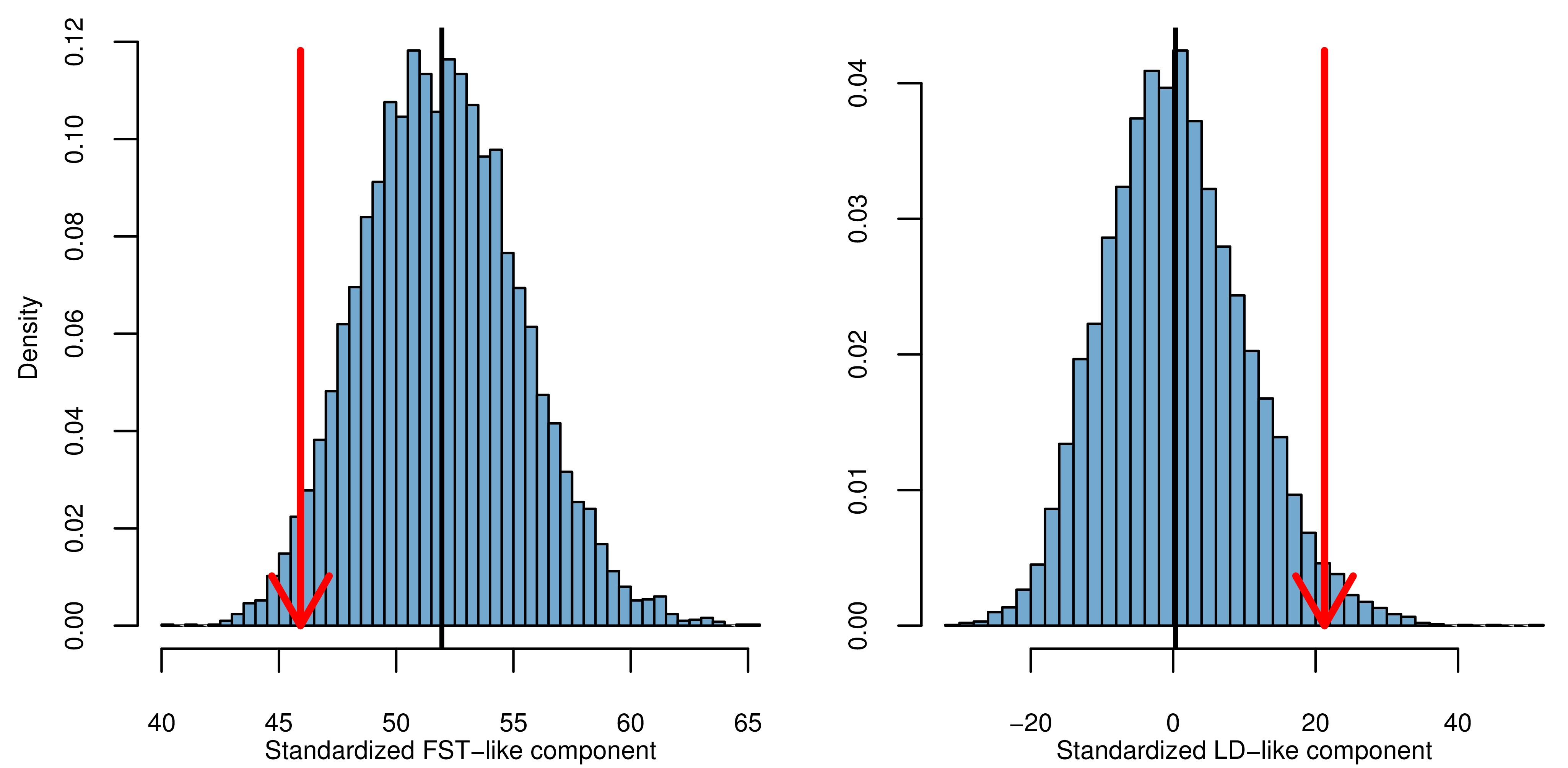

Supplement: Figure S7 — The two components of for the BMI dataset, as described by the left and right terms in (14). The null distribution of each component is shows as a histogram. The expected value is shown as a black bar, and the observed value as a red arrow. (TIFF) [file pgen.1004412.s007.tiff]

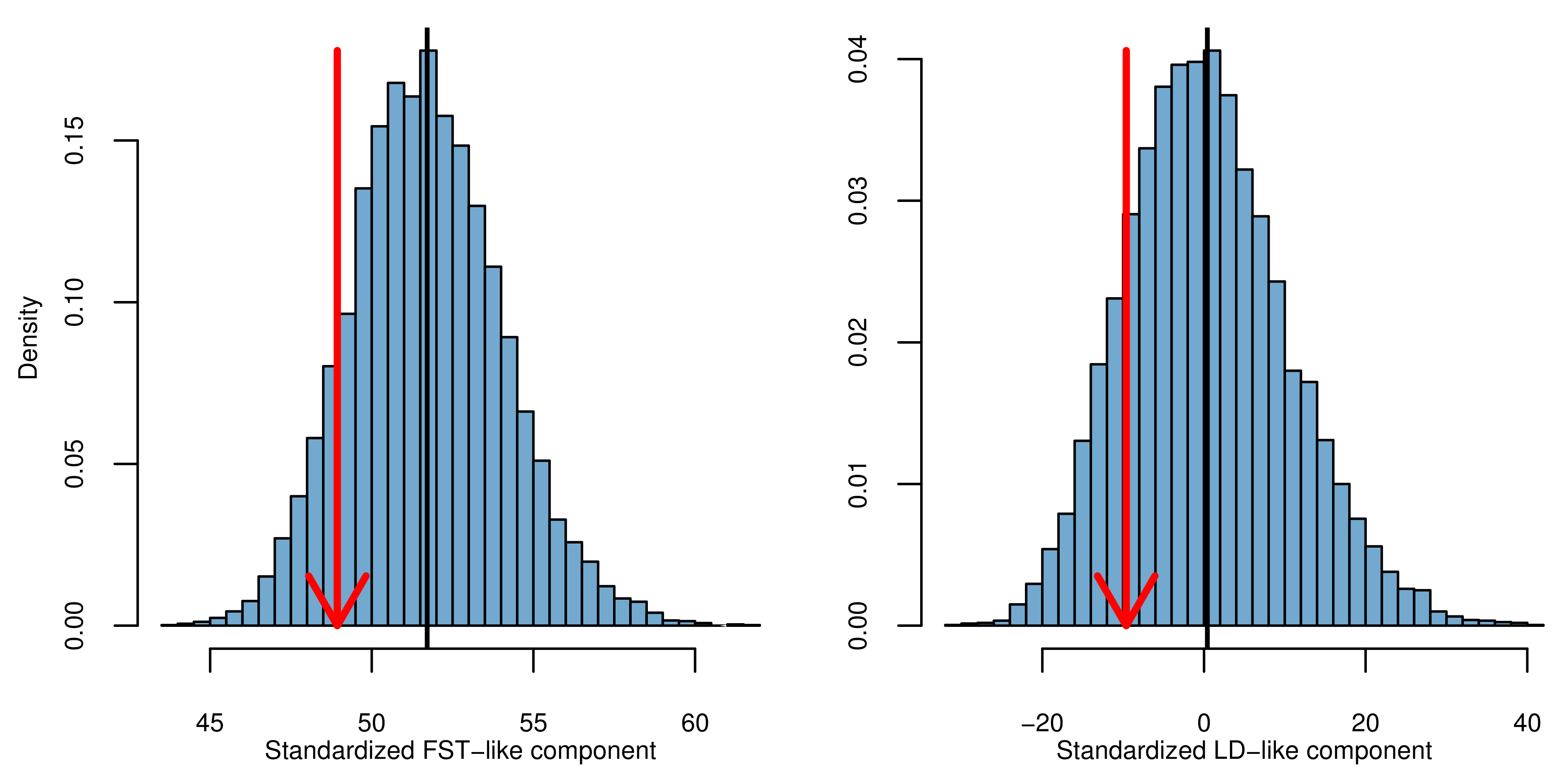

Supplement: Figure S8 — The two components of for the T2D dataset, as described by the left and right terms in (14). The null distribution of each component is shows as a histogram. The expected value is shown as a black bar, and the observed value as a red arrow. (TIFF) [file pgen.1004412.s008.tiff]

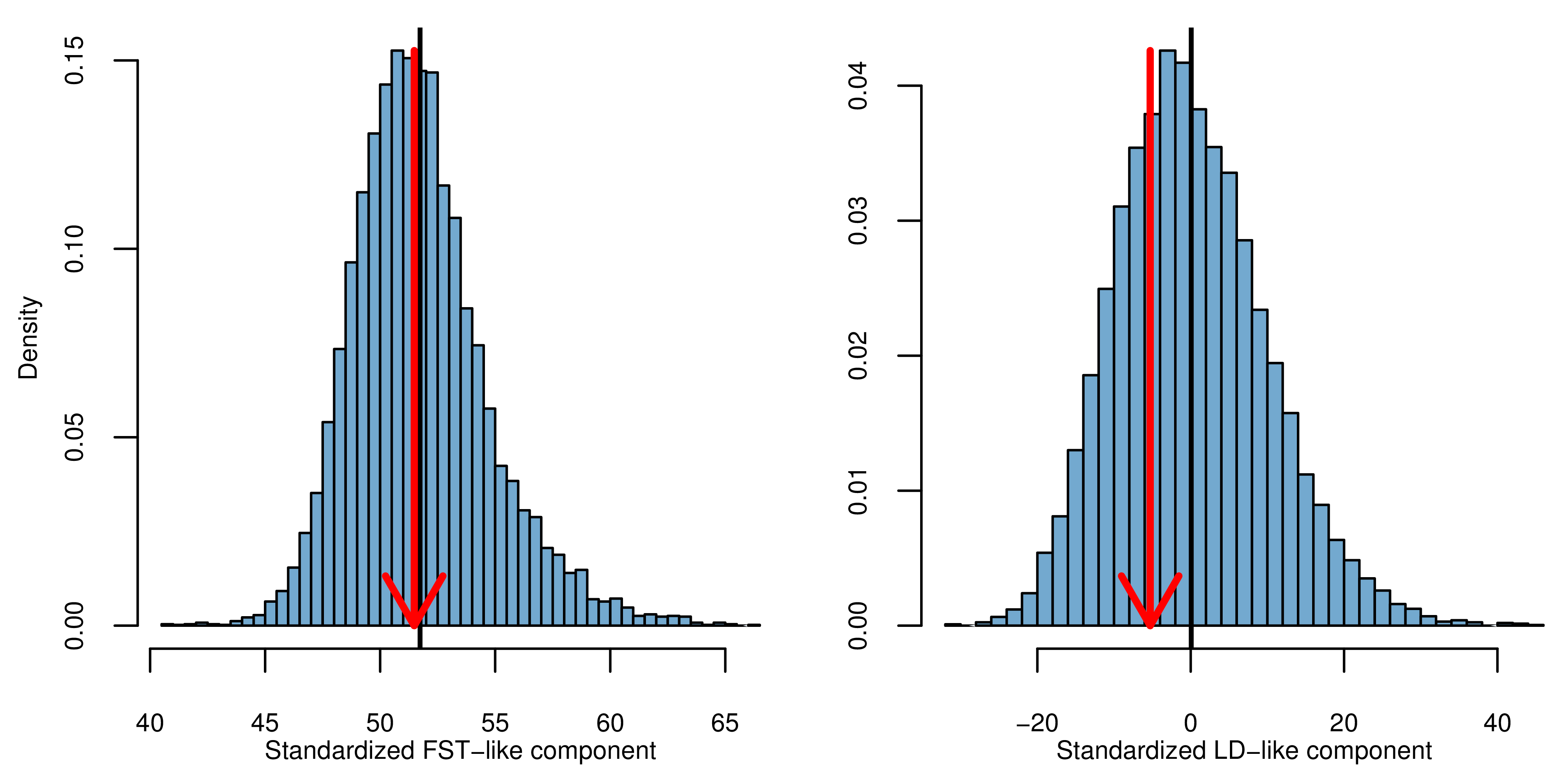

Supplement: Figure S9 — The two components of for the CD dataset, as described by the left and right terms in (14). The null distribution of each component is shows as a histogram. The expected value is shown as a black bar, and the observed value as a red arrow. (TIFF) [file pgen.1004412.s009.tiff]

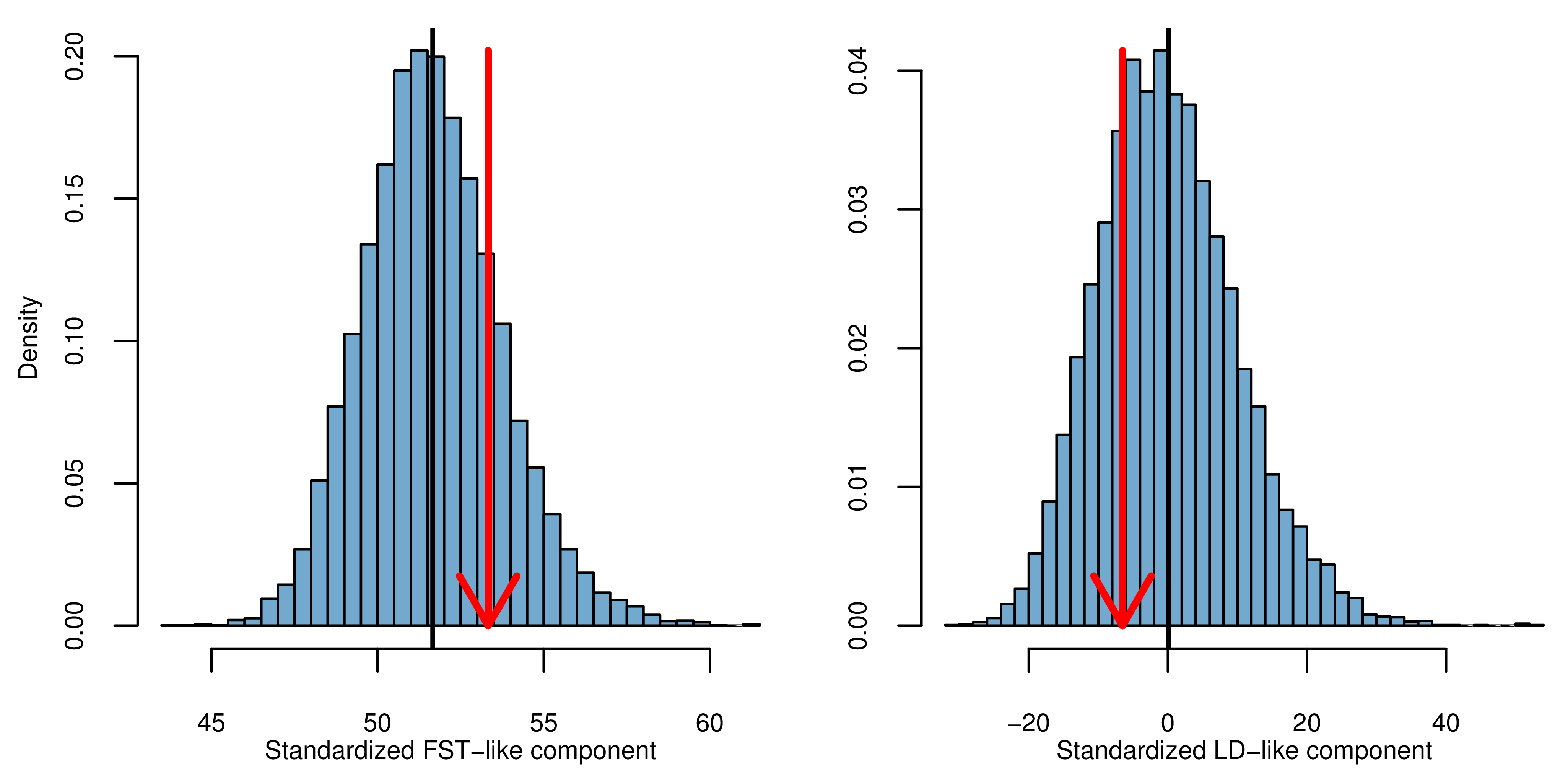

Supplement: Figure S10 — The two components of for the UC dataset, as described by the left and right terms in (14). The null distribution of each component is shows as a histogram. The expected value is shown as a black bar, and the observed value as a red arrow. (TIFF) [file pgen.1004412.s010.tiff]

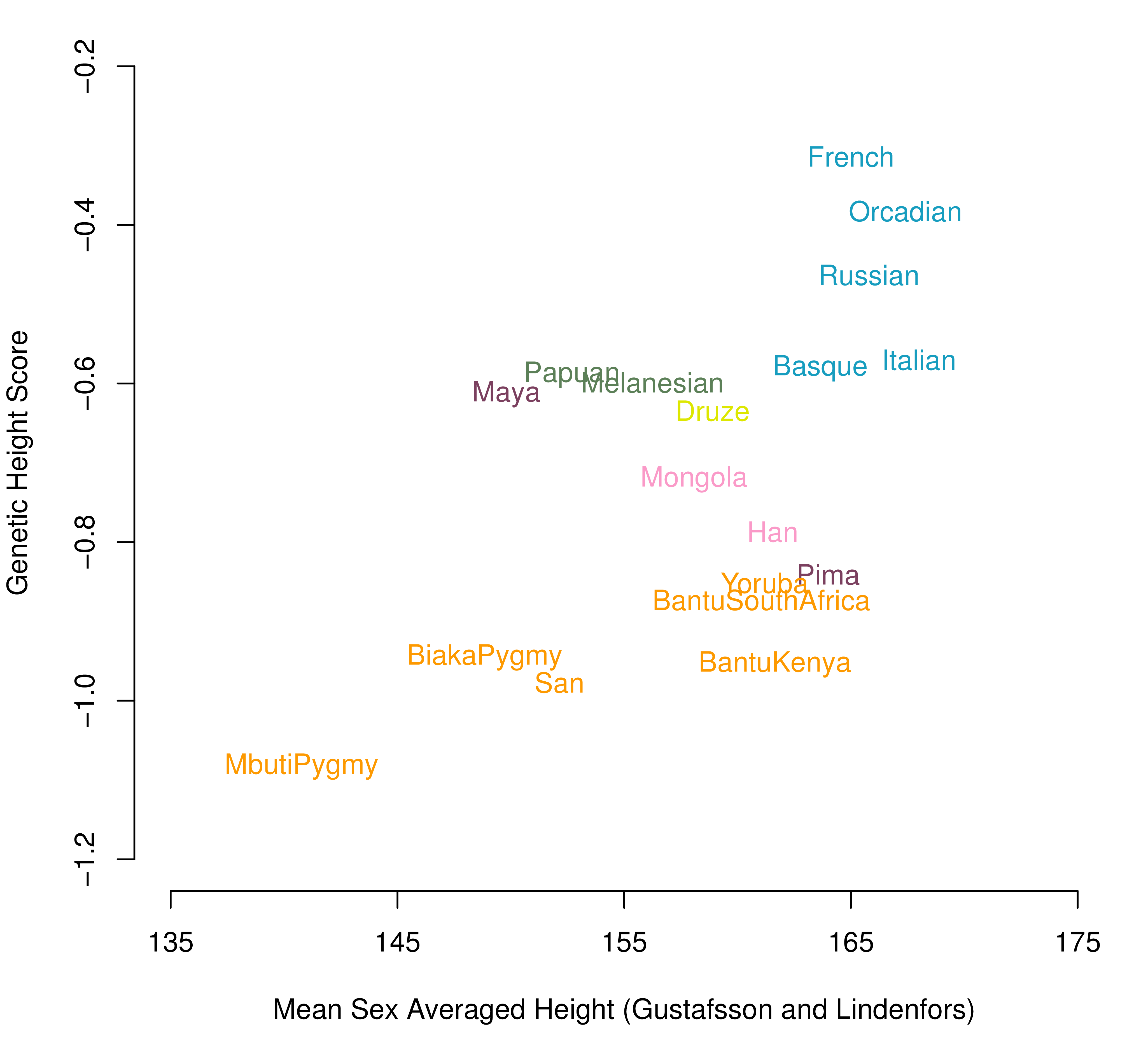

Supplement: Figure S11 — The genetic values for height in each HGDP population plotted against the measured sex averaged height taken from [127]. Only the subset of populations with an appropriately close match in the named population in [127]'s Appendix I are shown, values used are given in Supplementary table S1. (TIFF) [file pgen.1004412.s011.tiff]

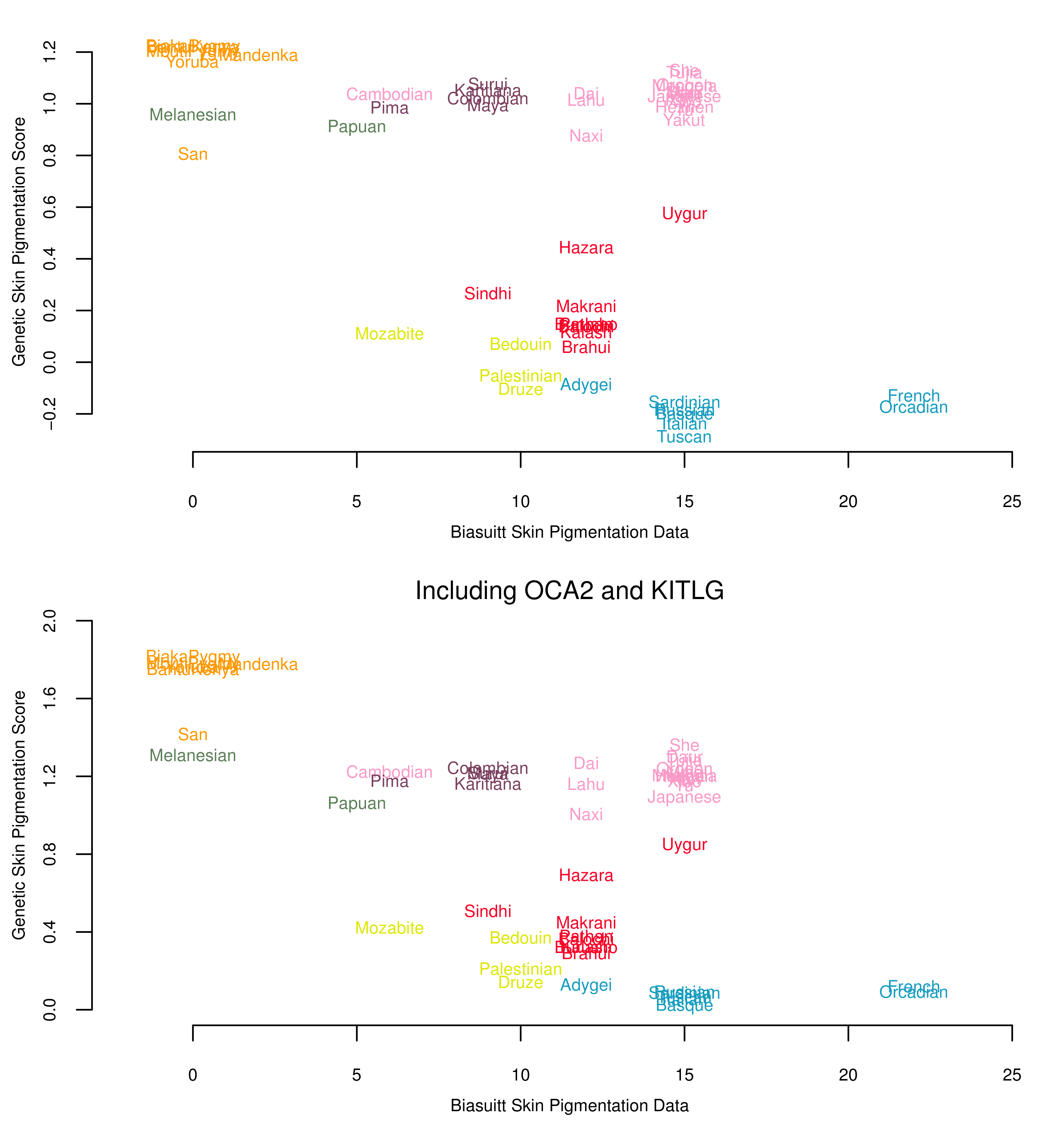

Supplement: Figure S12 — The genetic skin pigmentation score for a each HGDP population plotted against the HGDP populations values on the skin pigmentation index map of Biasutti 1959. Data obtained from Supplementary table of [69]. Note that Biasutti map is interpolated, and so values are known to be imperfect. Values used are given in Supplementary table S2. (TIFF) [file pgen.1004412.s012.tiff]

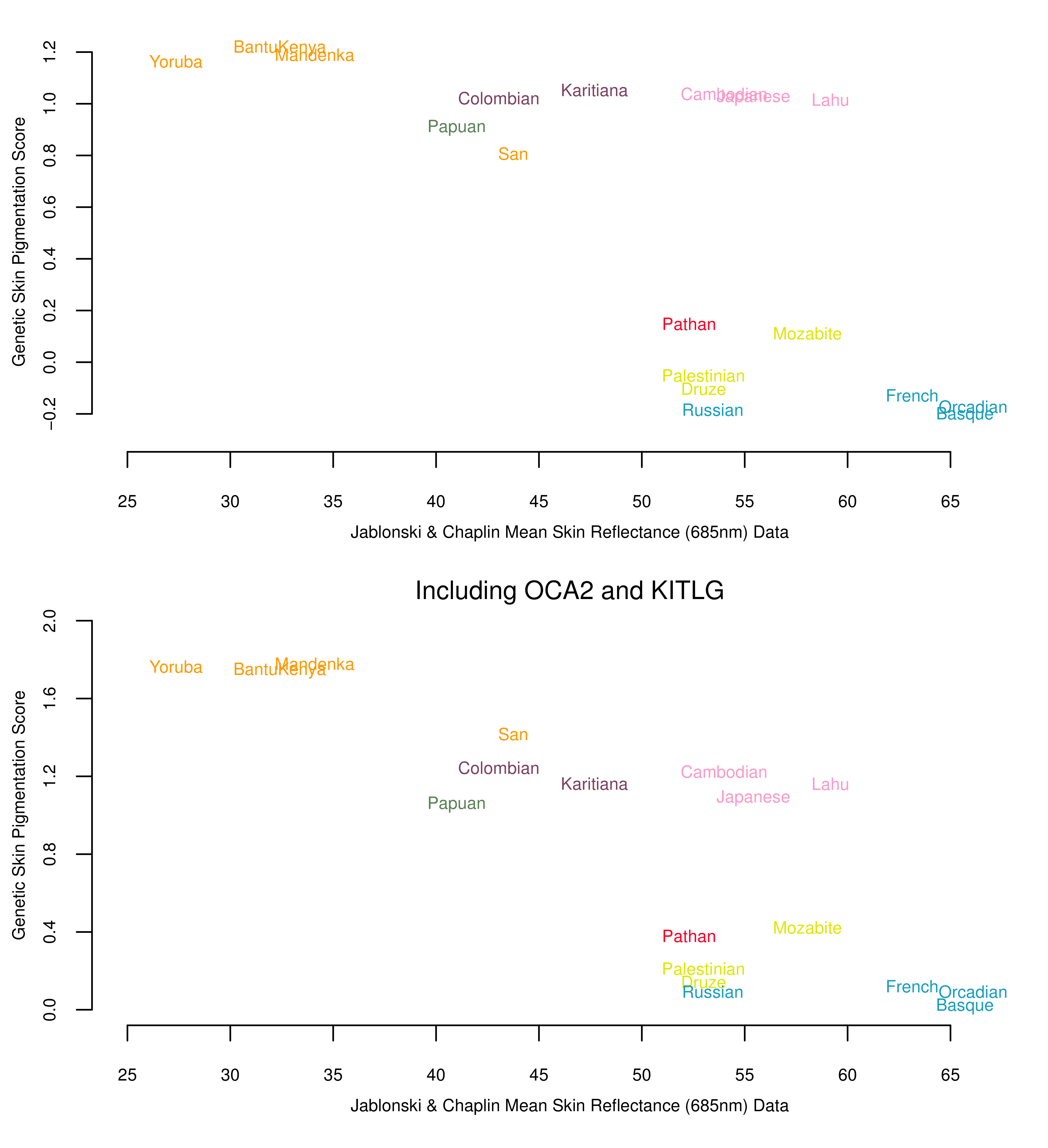

Supplement: Figure S13 — The genetic skin pigmentation score for a each HGDP population plotted against the HGDP populations values from the [68] mean skin reflectance (685nm) data (their Table 6). Only the subset of populations with an appropriately close match were used as in the Supplementary table of [69]. Values and populations used are given in Table S2. (TIFF) [file pgen.1004412.s013.tiff]

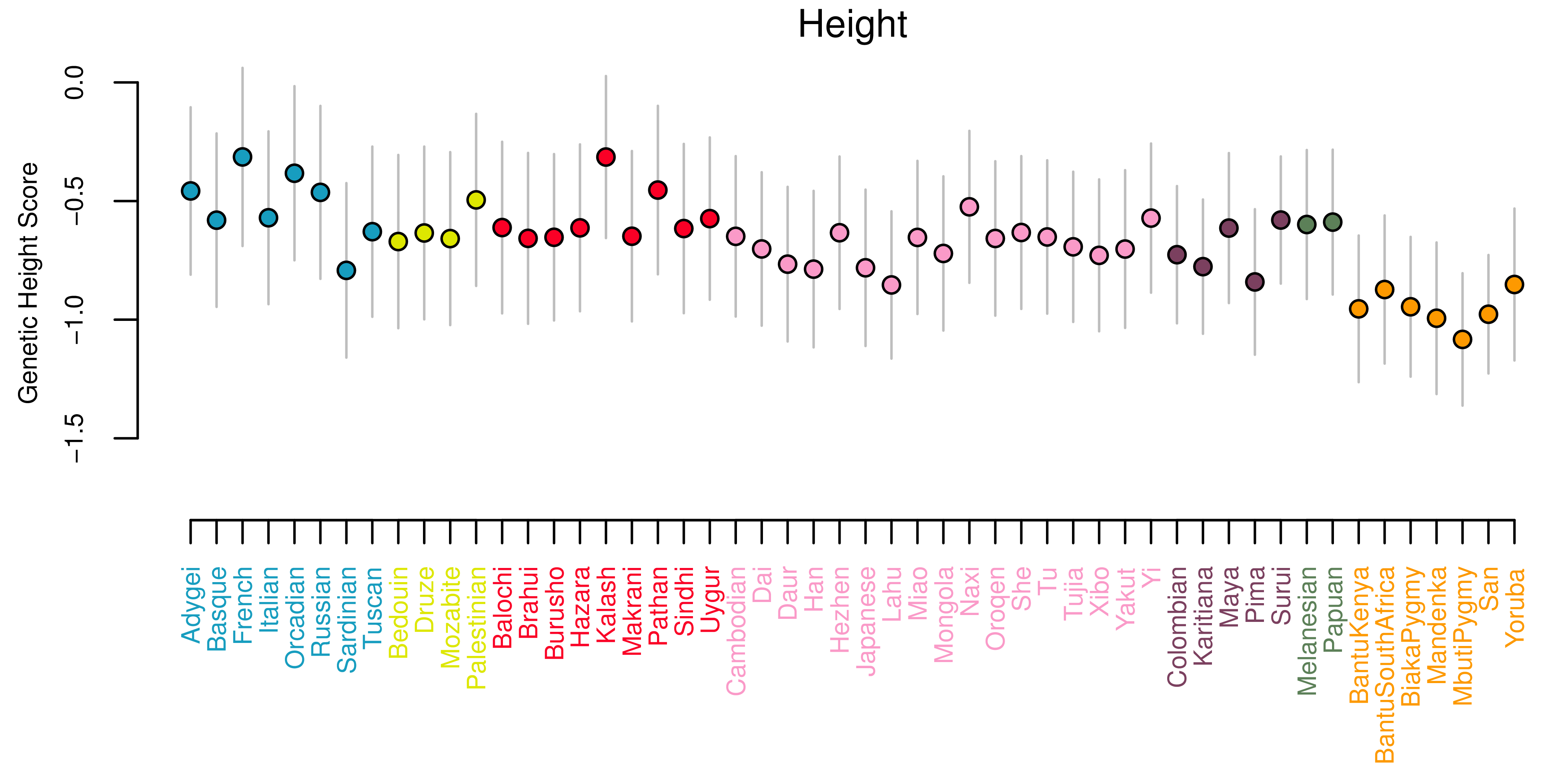

Supplement: Figure S14 — The distribution of genetic height score across all 52 HGDP populations. Grey bars represent the confidence interval for the genetic height score of an individual randomly chosen from that population under Hardy-Weinberg assumptions. (TIFF) [file pgen.1004412.s014.tiff]

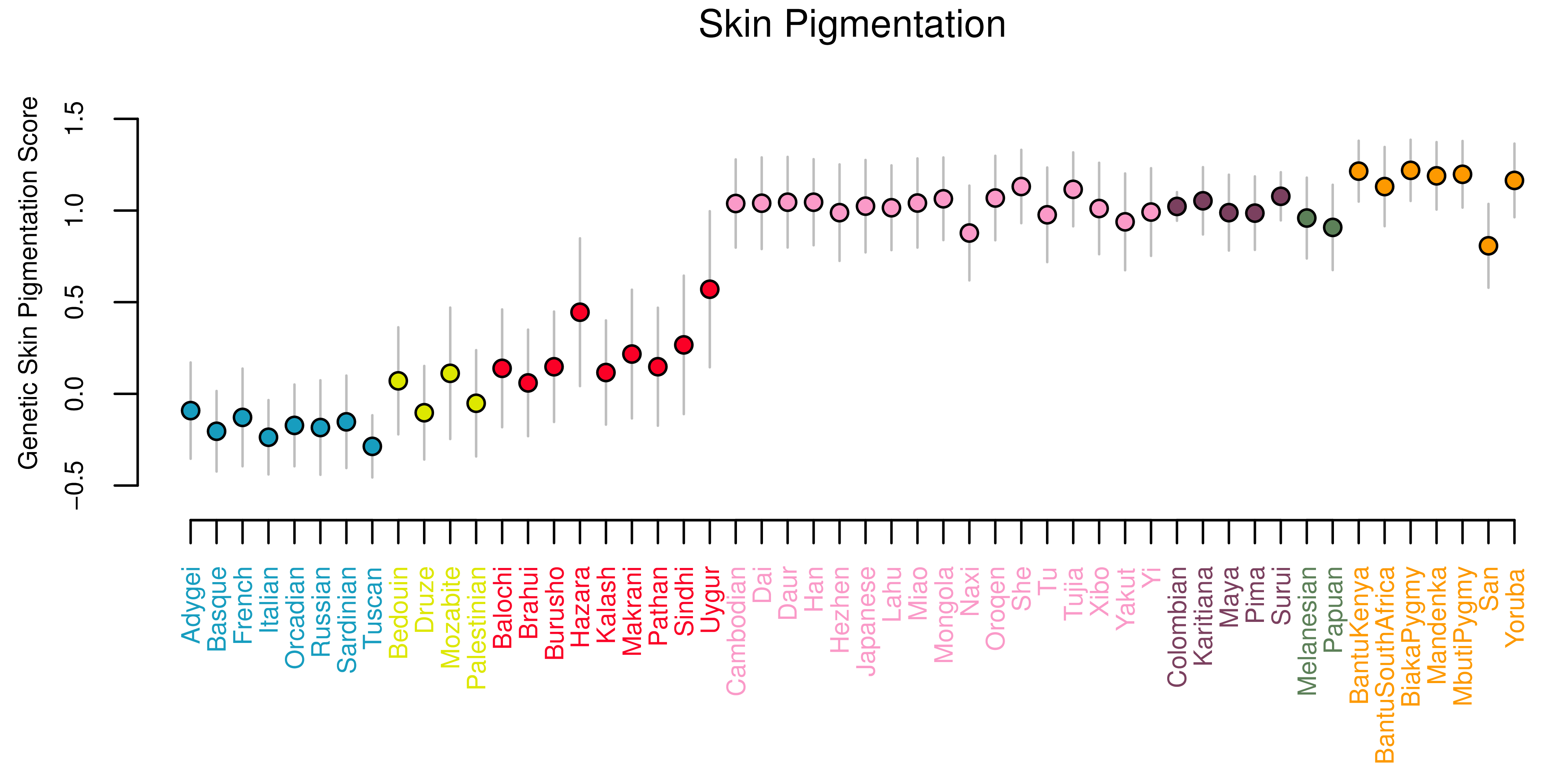

Supplement: Figure S15 — The distribution of genetic skin pigmentation score across all 52 HGDP populations. Grey bars represent the confidence interval for the genetic skin pigmentation score of an individual randomly chosen from that population under Hardy-Weinberg assumptions. (TIFF) [file pgen.1004412.s015.tiff]

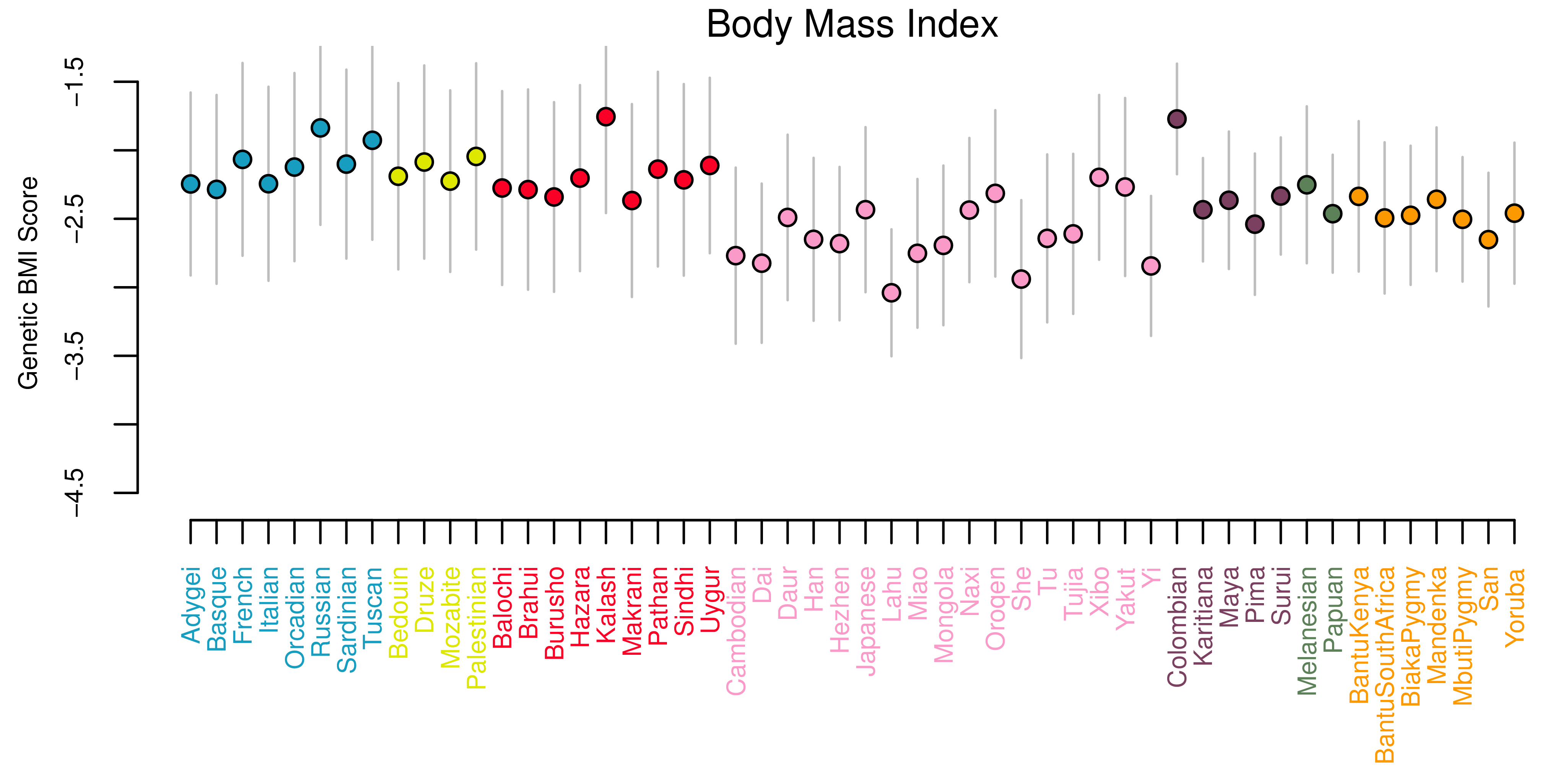

Supplement: Figure S16 — The distribution of genetic BMI score across all 52 HGDP populations. Grey bars represent the confidence interval for the genetic BMI score of an individual randomly chosen from that population under Hardy-Weinberg assumptions. (TIFF) [file pgen.1004412.s016.tiff]

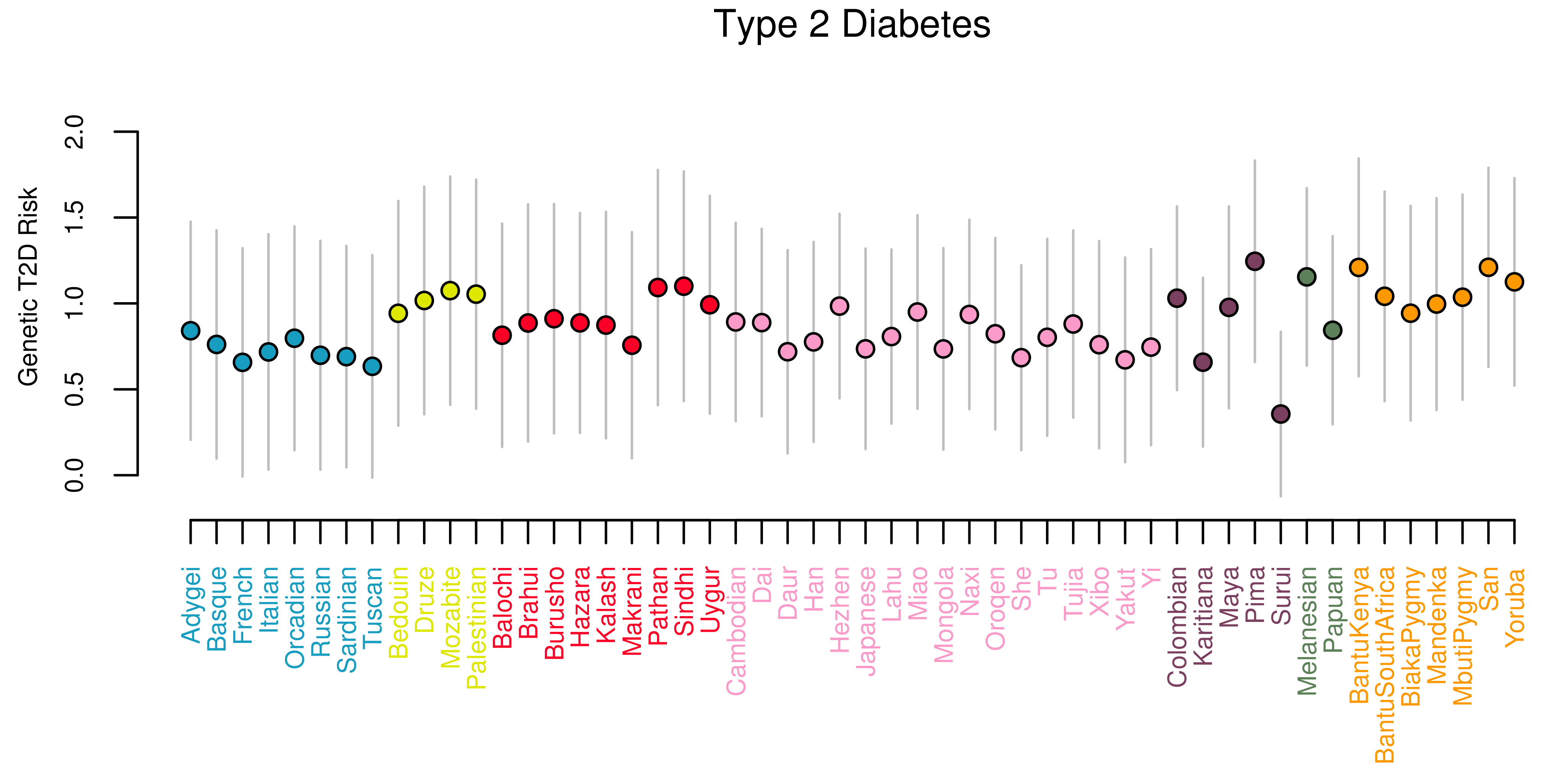

Supplement: Figure S17 — The distribution of genetic T2D risk score across all 52 HGDP populations. Grey bars represent the confidence interval for the genetic T2D risk score of an individual randomly chosen from that population under Hardy-Weinberg assumptions. (TIFF) [file pgen.1004412.s017.tiff]

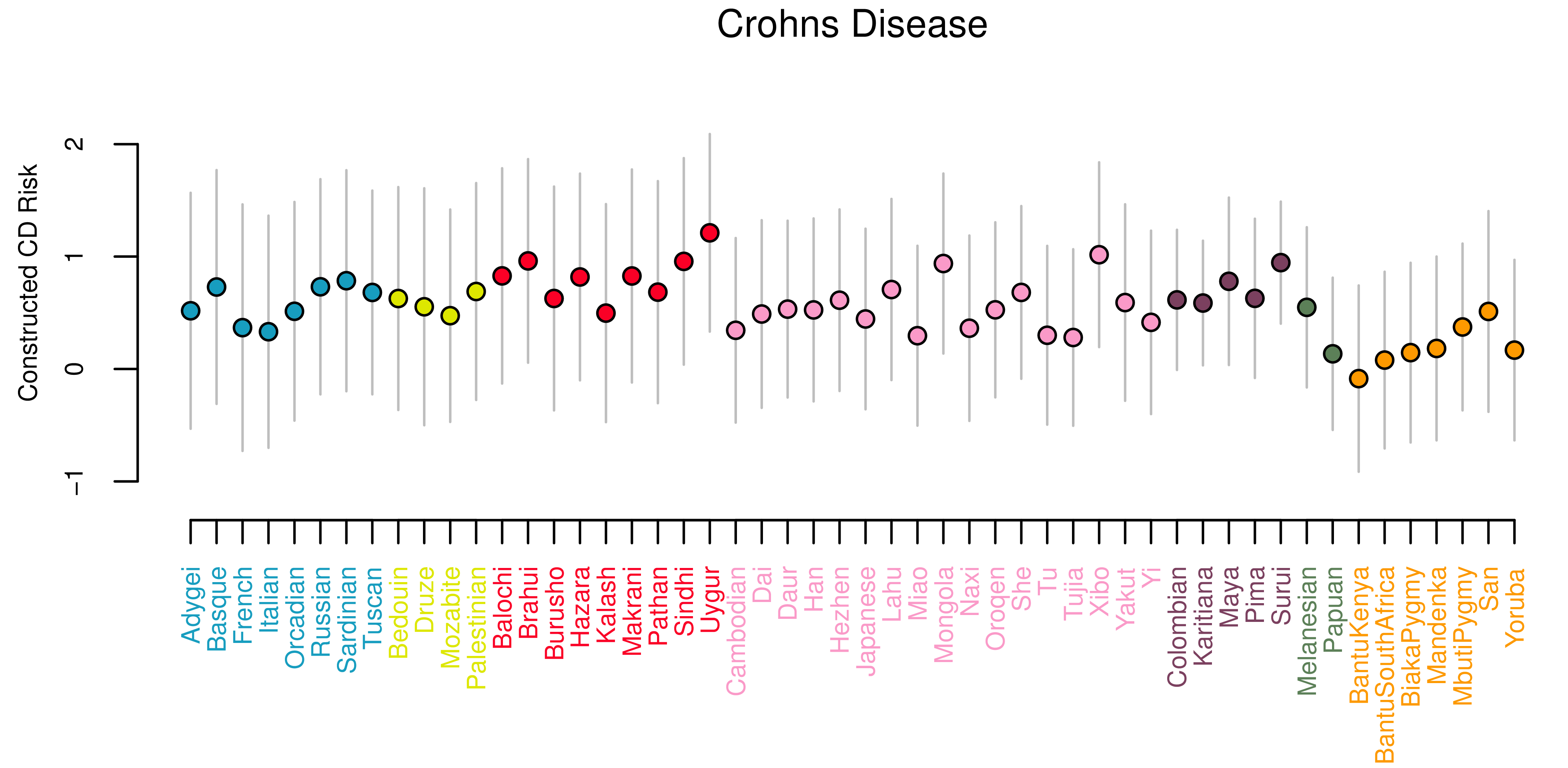

Supplement: Figure S18 — The distribution of genetic CD risk score across all 52 HGDP populations. Grey bars represent the confidence interval for the genetic CD risk score of an individual randomly chosen from that population under Hardy-Weinberg assumptions. (TIFF) [file pgen.1004412.s018.tiff]

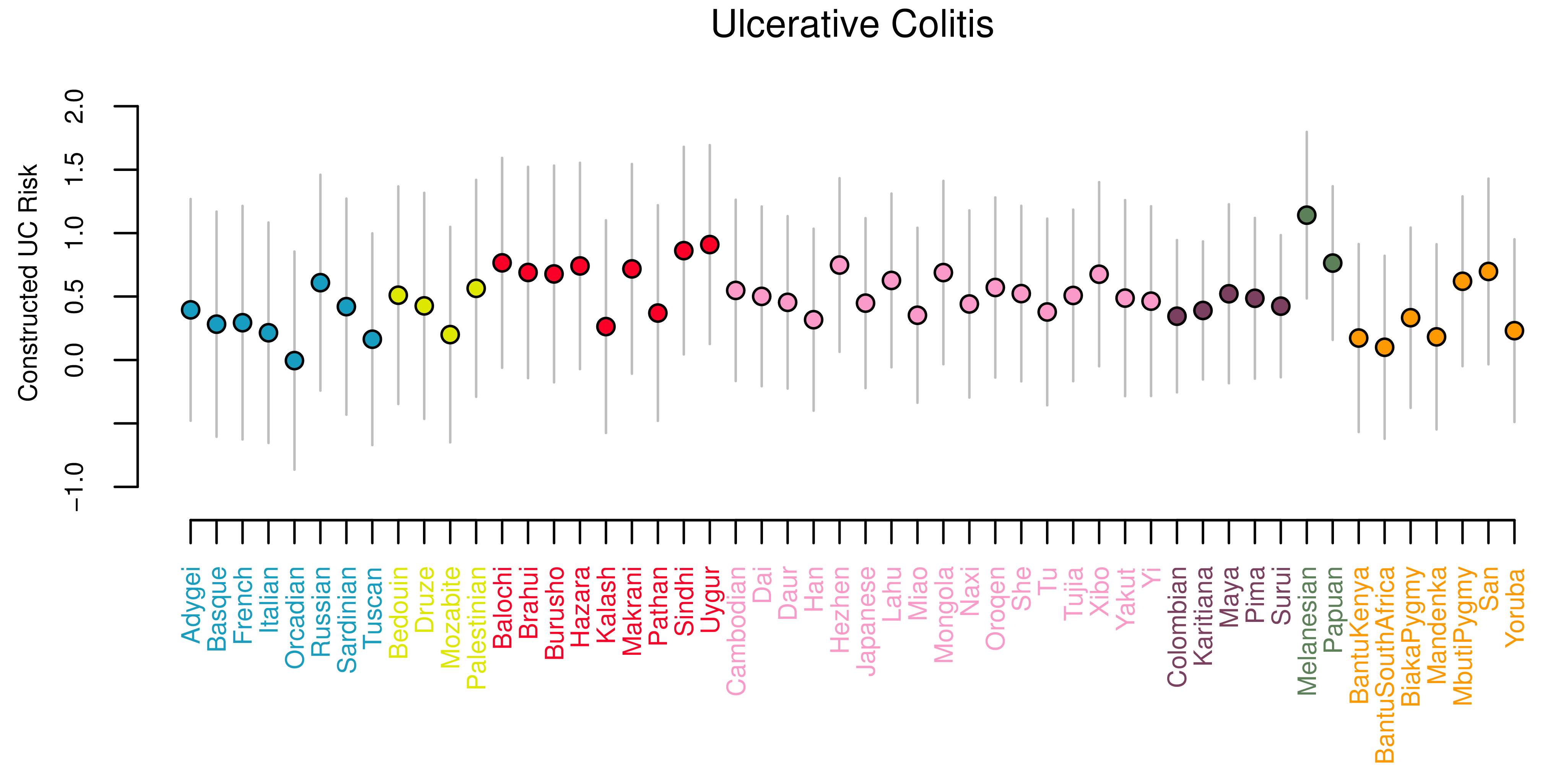

Supplement: Figure S19 — The distribution of genetic UC risk score across all 52 HGDP populations. Grey bars represent the confidence interval for the genetic UC risk score of an individual randomly chosen from that population under Hardy-Weinberg assumptions. (TIFF) [file pgen.1004412.s019.tiff]
