# Supplementary material for: A Population Genetic Signal of Polygenic Adaptation
Source: PLoS Genet. 2014 Aug 7;10(8):e1004412. doi: 10.1371/journal.pgen.1004412 (PMC4125079; doi:10.1371/journal.pgen.1004412)
Supplement: Table S1 — Genetic height scores as compared to true heights for populations with a suitably close match in the dataset of [127]. See Figure S11 for a plot of genetic height score against sex averaged height. (PDF) [file pgen.1004412.s020.pdf]

Population	Genetic Height Score	Male Height (cm)	Female Height (cm)	Sex Averaged Height (cm)
BantuKenya	-0.95	167.30	156.00	161.65
BantuSouthAfrica	-0.87	166.10	156.00	161.05
Basque	-0.58	170.00	157.30	163.65
BiakaPygmy	-0.95	152.70	145.00	148.85
Druze	-0.63	165.60	152.20	158.90
French	-0.31	169.60	160.40	165.00
Han	-0.79	167.10	156.00	161.55
Italian	-0.57	174.00	162.00	168.00
Maya	-0.61	156.80	142.80	149.80
MbutiPygmy	-1.08	144.19	137.35	140.77
Melanesian	-0.60	162.10	150.40	156.25
Mongola	-0.72	164.83	151.33	158.08
Orcadian	-0.38	173.90	160.90	167.40
Papuan	-0.59	155.97	149.41	152.69
Pima	-0.84	171.00	157.00	164.00
Russian	-0.46	171.80	159.80	165.80
San	-0.98	157.70	146.60	152.15
Yoruba	-0.85	167.50	155.00	161.25
