# Supplementary material for: A Population Genetic Signal of Polygenic Adaptation
Source: PLoS Genet. 2014 Aug 7;10(8):e1004412. doi: 10.1371/journal.pgen.1004412 (PMC4125079; doi:10.1371/journal.pgen.1004412)
Supplement: Table S2 — Genetic skin pigmentation score as compared to values from Biasutti [69], [128] and [68]. We also calculate a genetic skin pigmentation score including previously reported associations at KITLG and OCA2 for comparisson. See also Figures S12 and S13. (PDF) [file pgen.1004412.s021.pdf]

Population	Genetic Pigmentation Score	Genetic Pigment Score w/ KITLG+OCA2	Biasutti	Jablonski
Adygei	-0.09	0.12	12	
Balochi	0.14	0.36	12	
BantuKenya	1.21	1.74	0	32.40
Basque	-0.20	0.02	15	65.70
Bedouin	0.07	0.37	10	
BiakaPygmy	1.22	1.81	0	
Brahui	0.06	0.29	12	
Burusho	0.15	0.32	12	
Cambodian	1.04	1.22	6	54.00
Colombian	1.02	1.24	9	43.05
Dai	1.04	1.27	12	
Daur	1.05	1.30	15	
Druze	-0.10	0.14	10	53.00
French	-0.13	0.12	22	63.14
Han	1.05	1.19	15	
Hazara	0.45	0.69	12	
Hezhen	0.99	1.20	15	
Italian	-0.24	0.05	15	
Japanese	1.02	1.09	15	55.42
Kalash	0.12	0.33	12	
Karitiana	1.05	1.16	9	47.70
Lahu	1.01	1.16	12	59.17
Makrani	0.22	0.45	12	
Mandenka	1.19	1.78	2	34.10
Maya	0.99	1.21	9	
MbutiPygmy	1.20	1.77	0	
Melanesian	0.96	1.31	0	
Miao	1.04	1.22	15	
Mongola	1.06	1.20	15	
Mozabite	0.11	0.42	6	58.05
Naxi	0.88	1.01	12	
Orcadian	-0.17	0.09	22	66.10
Oroqen	1.07	1.23	15	
Palestinian	-0.05	0.21	10	53.00
Papuan	0.91	1.06	5	41.00
Pathan	0.15	0.38	12	52.30
Pima	0.99	1.18	6	
Russian	-0.18	0.09	15	53.45
San	0.81	1.42	0	43.75
Sardinian	-0.15	0.09	15	
She	1.13	1.36	15	
Sindhi	0.27	0.51	9	
Surui	1.08	1.22	9	
Tu	0.98	1.15	15	
Tujia	1.12	1.28	15	
Tuscan	-0.29	0.07	15	
Uygur	0.57	0.84	15	
Xibo	1.01	1.17	15	
Yakut	0.94	1.20	15	
Yi	0.99	1.19	15	
Yoruba	1.16	1.76	0	27.40
