# Supplementary material for: A Population Genetic Signal of Polygenic Adaptation
Source: PLoS Genet. 2014 Aug 7;10(8):e1004412. doi: 10.1371/journal.pgen.1004412 (PMC4125079; doi:10.1371/journal.pgen.1004412)
Supplement: Table S3 — Conditional analysis at the regional level for the height dataset. (PDF) [file pgen.1004412.s022.pdf]

	Observed	Expected	Variance	Z	p
Europe	-0.52	-0.56	0.0030	0.58	0.563573
Middle East	-0.61	-0.64	0.0025	0.50	0.620092
Central Asia	-0.57	-0.54	0.0025	-0.65	0.514631
East Asia	-0.69	-0.69	0.0068	0.01	0.990422
Americas	-0.71	-0.61	0.0234	-0.62	0.532068
Oceania	-0.59	-0.65	0.0312	0.31	0.757571
Africa	-0.95	-0.64	0.0238	-2.00	**0.045035**
