# Supplementary material for: A Population Genetic Signal of Polygenic Adaptation
Source: PLoS Genet. 2014 Aug 7;10(8):e1004412. doi: 10.1371/journal.pgen.1004412 (PMC4125079; doi:10.1371/journal.pgen.1004412)
Supplement: Table S4 — Conditional analysis at the individual population level for the height dataset. (PDF) [file pgen.1004412.s023.pdf]

	Observed	Expected	Variance	Z	p
Adygei	-0.46	-0.52	0.0058	0.81	0.420047
Balochi	-0.61	-0.59	0.0039	-0.34	0.732533
BantuKenya	-0.95	-0.88	0.0074	-0.89	0.370989
BantuSouthAfrica	-0.87	-0.93	0.0087	0.57	0.565944
Basque	-0.58	-0.49	0.0058	-1.14	0.253001
Bedouin	-0.67	-0.60	0.0040	-1.16	0.245140
BiakaPygmy	-0.95	-0.92	0.0089	-0.24	0.813609
Brahui	-0.66	-0.59	0.0041	-1.02	0.306021
Burusho	-0.65	-0.54	0.0049	-1.56	0.119344
Cambodian	-0.65	-0.72	0.0075	0.81	0.419708
Colombian	-0.73	-0.68	0.0149	-0.37	0.712734
Dai	-0.70	-0.72	0.0074	0.27	0.788165
Daur	-0.77	-0.71	0.0068	-0.73	0.466617
Druze	-0.63	-0.57	0.0039	-1.03	0.303758
French	-0.31	-0.55	0.0032	4.19	**0.000028**
Han	-0.79	-0.70	0.0018	-2.16	**0.030910**
Hazara	-0.61	-0.62	0.0040	0.09	0.932089
Hezhen	-0.63	-0.70	0.0070	0.79	0.431967
Italian	-0.57	-0.53	0.0067	-0.45	0.653982
Japanese	-0.78	-0.72	0.0036	-0.99	0.320879
Kalash	-0.31	-0.52	0.0110	1.92	0.054613
Karitiana	-0.78	-0.65	0.0205	-0.87	0.383564
Lahu	-0.85	-0.70	0.0113	-1.46	0.144720
Makrani	-0.65	-0.60	0.0038	-0.75	0.454499
Mandenka	-0.99	-0.88	0.0058	-1.45	0.146046
Maya	-0.61	-0.70	0.0070	1.05	0.295347
MbutiPygmy	-1.08	-0.95	0.0152	-1.12	0.262901
Melanesian	-0.60	-0.63	0.0199	0.24	0.810697
Miao	-0.65	-0.72	0.0065	0.84	0.398726
Mongola	-0.72	-0.69	0.0057	-0.43	0.664301
Mozabite	-0.66	-0.65	0.0071	-0.06	0.952256
Naxi	-0.52	-0.68	0.0088	1.67	0.095674
Orcadian	-0.38	-0.48	0.0069	1.20	0.228890
Oroqen	-0.66	-0.73	0.0075	0.85	0.397609
Palestinian	-0.50	-0.65	0.0031	2.82	**0.004789**
Papuan	-0.59	-0.59	0.0235	0.02	0.984319
Pathan	-0.45	-0.57	0.0042	1.86	0.063140
Pima	-0.84	-0.67	0.0152	-1.42	0.156243
Russian	-0.46	-0.47	0.0048	0.13	0.893316
San	-0.98	-0.95	0.0278	-0.16	0.873515
Sardinian	-0.79	-0.51	0.0053	-3.88	**0.000105**
She	-0.63	-0.72	0.0073	1.03	0.303926
Sindhi	-0.62	-0.57	0.0039	-0.69	0.487958
Surui	-0.58	-0.70	0.0253	0.78	0.433809
Tu	-0.65	-0.69	0.0062	0.48	0.630360
Tujia	-0.69	-0.72	0.0062	0.37	0.712043
Tuscan	-0.63	-0.52	0.0113	-1.00	0.318431
Uygur	-0.57	-0.59	0.0074	0.19	0.847180
Xibo	-0.73	-0.70	0.0068	-0.30	0.760787
Yakut	-0.70	-0.66	0.0059	-0.49	0.626665
Yi	-0.57	-0.71	0.0068	1.72	0.084613
Yoruba	-0.85	-0.96	0.0048	1.59	0.111605
