# Supplementary material for: A Population Genetic Signal of Polygenic Adaptation
Source: PLoS Genet. 2014 Aug 7;10(8):e1004412. doi: 10.1371/journal.pgen.1004412 (PMC4125079; doi:10.1371/journal.pgen.1004412)
Supplement: Table S5 — Conditional analysis at the regional level for the skin pigmentation dataset. (PDF) [file pgen.1004412.s024.pdf]

	Observed	Expected	Variance	Z	p
Europe	-0.18	-0.03	0.0030	-2.75	**0.005935**
Middle East	0.01	0.01	0.0030	-0.14	0.889313
Central Asia	0.23	0.25	0.0027	-0.24	0.813941
East Asia	1.02	0.82	0.0086	2.15	**0.031684**
Americas	1.03	0.68	0.0333	1.91	0.056218
Oceania	0.93	0.80	0.0392	0.68	0.493996
Africa	1.13	0.63	0.0506	2.21	**0.027041**
