# Supplementary material for: A Population Genetic Signal of Polygenic Adaptation
Source: PLoS Genet. 2014 Aug 7;10(8):e1004412. doi: 10.1371/journal.pgen.1004412 (PMC4125079; doi:10.1371/journal.pgen.1004412)
Supplement: Table S6 — Conditional analysis at the individual population level for the skin pigmentation dataset. (PDF) [file pgen.1004412.s025.pdf]

	Observed	Exptected	Variance	Z	p
Adygei	-0.09	-0.04	0.0062	-0.64	0.525090
Balochi	0.14	0.13	0.0046	0.20	0.838807
BantuKenya	1.21	1.08	0.0128	1.16	0.247685
BantuSouthAfrica	1.13	1.17	0.0144	-0.32	0.746846
Basque	-0.20	-0.17	0.0063	-0.38	0.704915
Bedouin	0.07	-0.05	0.0049	1.74	0.081934
BiakaPygmy	1.22	1.11	0.0143	0.93	0.350280
Brahui	0.06	0.13	0.0050	-0.98	0.326220
Burusho	0.15	0.26	0.0057	-1.53	0.125199
Cambodian	1.04	0.92	0.0096	1.16	0.244370
Colombian	1.02	1.10	0.0188	-0.59	0.553594
Dai	1.04	1.04	0.0100	0.04	0.965427
Daur	1.05	0.99	0.0091	0.61	0.543435
Druze	-0.10	-0.09	0.0045	-0.20	0.844204
French	-0.13	-0.20	0.0039	1.22	0.222146
Han	1.05	1.06	0.0026	-0.33	0.744919
Hazara	0.45	0.49	0.0052	-0.64	0.519429
Hezhen	0.99	1.05	0.0100	-0.64	0.519743
Italian	-0.24	-0.12	0.0082	-1.26	0.207688
Japanese	1.02	1.06	0.0048	-0.54	0.586193
Kalash	0.12	0.09	0.0134	0.21	0.833512
Karitiana	1.05	1.07	0.0254	-0.10	0.919046
Lahu	1.02	1.03	0.0143	-0.09	0.924466
Makrani	0.22	0.14	0.0049	1.17	0.240751
Mandenka	1.19	1.12	0.0102	0.64	0.525346
Maya	0.99	0.85	0.0088	1.43	0.151325
MbutiPygmy	1.20	1.12	0.0252	0.50	0.616103
Melanesian	0.96	0.87	0.0284	0.51	0.611448
Miao	1.04	1.06	0.0087	-0.25	0.803925
Mongola	1.06	0.96	0.0079	1.17	0.240282
Mozabite	0.11	0.19	0.0096	-0.81	0.416292
Naxi	0.88	1.02	0.0112	-1.32	0.185939
Orcadian	-0.17	-0.12	0.0078	-0.60	0.548105
Oroqen	1.07	1.02	0.0098	0.49	0.626438
Palestinian	-0.05	0.02	0.0036	-1.16	0.247433
Papuan	0.91	0.89	0.0326	0.10	0.919411
Pathan	0.15	0.16	0.0048	-0.11	0.911081
Pima	0.99	0.97	0.0200	0.10	0.923266
Russian	-0.18	-0.05	0.0053	-1.89	0.058819
San	0.81	1.07	0.0405	-1.31	0.191477
Sardinian	-0.15	-0.20	0.0062	0.55	0.583092
She	1.13	1.04	0.0095	0.94	0.345316
Sindhi	0.27	0.22	0.0052	0.67	0.501326
Surui	1.08	0.99	0.0365	0.45	0.652715
Tu	0.98	0.99	0.0086	-0.12	0.905437
Tujia	1.12	1.07	0.0083	0.54	0.585802
Tuscan	-0.29	-0.13	0.0128	-1.36	0.174556
Uygur	0.57	0.50	0.0085	0.73	0.465493
Xibo	1.01	0.96	0.0086	0.56	0.577734
Yakut	0.94	0.86	0.0078	0.88	0.377402
Yi	0.99	1.02	0.0087	-0.32	0.745359
Yoruba	1.16	1.23	0.0086	-0.66	0.508211
