# Supplementary material for: A Population Genetic Signal of Polygenic Adaptation
Source: PLoS Genet. 2014 Aug 7;10(8):e1004412. doi: 10.1371/journal.pgen.1004412 (PMC4125079; doi:10.1371/journal.pgen.1004412)
Supplement: Table S7 — Condtional analysis at the regional level for the BMI dataset. (PDF) [file pgen.1004412.s026.pdf]

	Observed	Expected	Variance	Z	p
Europe	-2.10	-2.10	0.0092	-0.02	0.986166
Middle East	-2.14	-2.17	0.0080	0.41	0.683249
Central Asia	-2.19	-2.24	0.0077	0.63	0.528510
East Asia	-2.62	-2.24	0.0224	-2.53	**0.011480**
Americas	-2.29	-2.35	0.0788	0.21	0.832973
Oceania	-2.36	-2.51	0.1036	0.48	0.632885
Africa	-2.47	-2.37	0.0826	-0.33	0.742233
