# Supplementary material for: A Population Genetic Signal of Polygenic Adaptation
Source: PLoS Genet. 2014 Aug 7;10(8):e1004412. doi: 10.1371/journal.pgen.1004412 (PMC4125079; doi:10.1371/journal.pgen.1004412)
Supplement: Table S8 — Conditional analysis at the individual population level for the BMI dataset. (PDF) [file pgen.1004412.s027.pdf]

	Observed	Expected	Variance	Z	p
Adygei	-2.25	-2.09	0.0193	-1.13	0.259383
Balochi	-2.28	-2.26	0.0124	-0.12	0.906459
BantuKenya	-2.34	-2.45	0.0252	0.71	0.475069
BantuSouthAfrica	-2.49	-2.43	0.0297	-0.35	0.724172
Basque	-2.29	-2.07	0.0182	-1.59	0.110893
Bedouin	-2.19	-2.11	0.0128	-0.68	0.493665
BiakaPygmy	-2.47	-2.47	0.0290	-0.05	0.959030
Brahui	-2.29	-2.22	0.0143	-0.55	0.582714
Burusho	-2.34	-2.18	0.0147	-1.33	0.183764
Cambodian	-2.77	-2.73	0.0247	-0.27	0.788781
Colombian	-1.77	-2.44	0.0498	2.98	**0.002842**
Dai	-2.82	-2.73	0.0248	-0.60	0.545319
Daur	-2.49	-2.51	0.0237	0.11	0.913165
Druze	-2.09	-2.15	0.0128	0.58	0.561551
French	-2.07	-2.09	0.0104	0.20	0.844786
Han	-2.65	-2.65	0.0061	0.06	0.951026
Hazara	-2.20	-2.32	0.0124	1.04	0.300183
Hezhen	-2.68	-2.47	0.0241	-1.39	0.165463
Italian	-2.24	-2.09	0.0220	-1.06	0.287855
Japanese	-2.43	-2.59	0.0118	1.49	0.137107
Kalash	-1.75	-2.19	0.0364	2.28	**0.022595**
Karitiana	-2.43	-2.37	0.0680	-0.25	0.801202
Lahu	-3.04	-2.68	0.0378	-1.86	0.063279
Makrani	-2.37	-2.21	0.0122	-1.46	0.143943
Mandenka	-2.36	-2.44	0.0193	0.61	0.540442
Maya	-2.36	-2.20	0.0224	-1.12	0.264309
MbutiPygmy	-2.50	-2.49	0.0536	-0.07	0.947811
Melanesian	-2.25	-2.44	0.0676	0.74	0.459264
Miao	-2.75	-2.69	0.0218	-0.40	0.689780
Mongola	-2.69	-2.43	0.0192	-1.92	0.054866
Mozabite	-2.23	-2.15	0.0247	-0.49	0.625131
Naxi	-2.44	-2.70	0.0288	1.56	0.119884
Orcadian	-2.12	-2.07	0.0215	-0.37	0.709254
Oroqen	-2.31	-2.50	0.0241	1.18	0.237730
Palestinian	-2.04	-2.16	0.0097	1.13	0.258466
Papuan	-2.46	-2.37	0.0791	-0.33	0.743669
Pathan	-2.14	-2.21	0.0135	0.64	0.523054
Pima	-2.54	-2.30	0.0489	-1.10	0.271635
Russian	-1.84	-2.13	0.0151	2.37	**0.017852**
San	-2.65	-2.47	0.0894	-0.62	0.535534
Sardinian	-2.10	-2.12	0.0166	0.11	0.910561
She	-2.94	-2.70	0.0245	-1.54	0.123110
Sindhi	-2.22	-2.25	0.0135	0.33	0.741178
Surui	-2.33	-2.26	0.0834	-0.24	0.808288
Tu	-2.64	-2.57	0.0206	-0.52	0.604447
Tujia	-2.61	-2.63	0.0201	0.16	0.875686
Tuscan	-1.93	-2.13	0.0355	1.10	0.273049
Uygur	-2.11	-2.34	0.0228	1.54	0.124610
Xibo	-2.20	-2.50	0.0212	2.07	**0.038059**
Yakut	-2.27	-2.32	0.0192	0.40	0.690521
Yi	-2.84	-2.59	0.0219	-1.73	0.082850
Yoruba	-2.46	-2.38	0.0170	-0.61	0.541470
