# Supplementary material for: A Population Genetic Signal of Polygenic Adaptation
Source: PLoS Genet. 2014 Aug 7;10(8):e1004412. doi: 10.1371/journal.pgen.1004412 (PMC4125079; doi:10.1371/journal.pgen.1004412)
Supplement: Table S9 — Conditional analysis at the regional level for the T2D dataset. (PDF) [file pgen.1004412.s028.pdf]

	Observed	Expected	Variance	Z	p
Europe	0.72	1.00	0.0097	-2.79	**0.005315**
Middle East	1.02	0.81	0.0079	2.37	**0.017671**
Central Asia	0.92	0.88	0.0078	0.53	0.598600
East Asia	0.81	0.98	0.0216	-1.16	0.244114
Americas	0.85	0.79	0.0773	0.21	0.831384
Oceania	1.00	0.96	0.1013	0.11	0.912009
Africa	1.08	1.11	0.0813	-0.10	0.918661
