# Supplementary material for: A Population Genetic Signal of Polygenic Adaptation
Source: PLoS Genet. 2014 Aug 7;10(8):e1004412. doi: 10.1371/journal.pgen.1004412 (PMC4125079; doi:10.1371/journal.pgen.1004412)
Supplement: Table S10 — Conditional analysis at the individual population level for the T2D dataset. (PDF) [file pgen.1004412.s029.pdf]

	Observed	Expected	Variance	Z	p
Adygei	0.84	0.88	0.0185	-0.30	0.763788
Balochi	0.81	0.93	0.0126	-1.02	0.306287
BantuKenya	1.21	1.05	0.0262	1.00	0.318499
BantuSouthAfrica	1.04	1.09	0.0300	-0.28	0.780539
Basque	0.76	0.72	0.0190	0.28	0.777993
Bedouin	0.94	0.99	0.0131	-0.39	0.694506
BiakaPygmy	0.94	1.09	0.0299	-0.82	0.409850
Brahui	0.89	0.92	0.0135	-0.27	0.784720
Burusho	0.91	0.93	0.0163	-0.16	0.875229
Cambodian	0.89	0.86	0.0254	0.22	0.829257
Colombian	1.03	0.88	0.0499	0.68	0.499559
Dai	0.89	0.82	0.0245	0.42	0.678026
Daur	0.72	0.79	0.0227	-0.49	0.627610
Druze	1.02	0.90	0.0130	1.06	0.291242
French	0.66	0.79	0.0110	-1.24	0.213762
Han	0.78	0.83	0.0063	-0.68	0.494587
Hazara	0.89	0.86	0.0134	0.25	0.799840
Hezhen	0.98	0.77	0.0256	1.37	0.172155
Italian	0.72	0.79	0.0223	-0.51	0.610084
Japanese	0.74	0.81	0.0124	-0.66	0.509728
Kalash	0.87	0.94	0.0380	-0.31	0.753570
Karitiana	0.66	0.87	0.0681	-0.81	0.419843
Lahu	0.81	0.83	0.0383	-0.11	0.909731
Makrani	0.76	0.94	0.0125	-1.66	0.097230
Mandenka	1.00	1.11	0.0203	-0.80	0.425733
Maya	0.98	0.90	0.0230	0.51	0.609012
MbutiPygmy	1.04	1.03	0.0518	0.04	0.971663
Melanesian	1.15	0.83	0.0695	1.24	0.215185
Miao	0.95	0.80	0.0225	0.99	0.322573
Mongola	0.74	0.79	0.0197	-0.42	0.676445
Mozabite	1.07	0.91	0.0239	1.08	0.279820
Naxi	0.94	0.80	0.0284	0.79	0.431243
Orcadian	0.80	0.73	0.0219	0.45	0.655908
Oroqen	0.82	0.79	0.0239	0.24	0.812587
Palestinian	1.05	0.89	0.0101	1.60	0.110698
Papuan	0.84	1.16	0.0802	-1.13	0.260430
Pathan	1.09	0.89	0.0138	1.74	0.081439
Pima	1.25	0.88	0.0543	1.57	0.116103
Russian	0.70	0.76	0.0158	-0.52	0.601197
San	1.21	0.98	0.0869	0.79	0.432263
Sardinian	0.69	0.82	0.0174	-0.99	0.322545
She	0.68	0.81	0.0246	-0.83	0.408294
Sindhi	1.10	0.92	0.0133	1.59	0.112277
Surui	0.36	0.95	0.0870	-2.01	**0.044245**
Tu	0.80	0.78	0.0216	0.15	0.877641
Tujia	0.88	0.81	0.0200	0.46	0.642326
Tuscan	0.63	0.85	0.0362	-1.11	0.265107
Uygur	0.99	0.84	0.0236	1.00	0.318441
Xibo	0.76	0.80	0.0223	-0.30	0.765435
Yakut	0.67	0.83	0.0208	-1.12	0.263127
Yi	0.75	0.83	0.0221	-0.56	0.573343
Yoruba	1.13	1.08	0.0168	0.33	0.740815
