# Supplementary material for: A Population Genetic Signal of Polygenic Adaptation
Source: PLoS Genet. 2014 Aug 7;10(8):e1004412. doi: 10.1371/journal.pgen.1004412 (PMC4125079; doi:10.1371/journal.pgen.1004412)
Supplement: Table S11 — Conditional analysis at the regional level for the CD dataset. (PDF) [file pgen.1004412.s030.pdf]

	Observed	Expected	Variance	Z	p
Europe	0.58	0.75	0.0188	-1.22	0.220764
Middle East	0.59	0.62	0.0166	-0.30	0.763396
Central Asia	0.82	0.55	0.0151	2.21	**0.027100**
East Asia	0.53	0.94	0.0459	-1.90	0.056975
Americas	0.71	0.67	0.1529	0.09	0.925298
Oceania	0.34	0.59	0.1959	-0.55	0.581088
Africa	0.20	0.52	0.1535	-0.83	0.409211
