# Supplementary material for: A Population Genetic Signal of Polygenic Adaptation
Source: PLoS Genet. 2014 Aug 7;10(8):e1004412. doi: 10.1371/journal.pgen.1004412 (PMC4125079; doi:10.1371/journal.pgen.1004412)
Supplement: Table S12 — Conditional analysis at the individual population level for the CD dataset. (PDF) [file pgen.1004412.s031.pdf]

	Observed	Expected	Variance	Z	p
Adygei	0.52	0.69	0.0378	-0.89	0.370810
Balochi	0.83	0.79	0.0236	0.22	0.823291
BantuKenya	-0.09	0.20	0.0485	-1.31	0.191212
BantuSouthAfrica	0.08	0.21	0.0566	-0.56	0.575256
Basque	0.73	0.57	0.0381	0.80	0.423331
Bedouin	0.63	0.62	0.0254	0.05	0.963091
BiakaPygmy	0.15	0.28	0.0574	-0.54	0.587432
Brahui	0.96	0.80	0.0273	0.97	0.332305
Burusho	0.63	0.74	0.0315	-0.61	0.538809
Cambodian	0.34	0.51	0.0500	-0.72	0.470030
Colombian	0.61	0.76	0.0986	-0.46	0.647213
Dai	0.49	0.46	0.0475	0.14	0.892361
Daur	0.53	0.63	0.0457	-0.47	0.637409
Druze	0.55	0.68	0.0254	-0.79	0.430724
French	0.37	0.67	0.0219	-2.04	**0.041575**
Han	0.53	0.52	0.0123	0.05	0.957247
Hazara	0.82	0.68	0.0266	0.84	0.401314
Hezhen	0.61	0.58	0.0494	0.16	0.870215
Italian	0.33	0.65	0.0436	-1.51	0.132112
Japanese	0.44	0.59	0.0239	-0.94	0.349319
Kalash	0.50	0.76	0.0726	-0.97	0.332454
Karitiana	0.59	0.77	0.1389	-0.50	0.615762
Lahu	0.71	0.42	0.0765	1.02	0.305499
Makrani	0.83	0.77	0.0247	0.34	0.734710
Mandenka	0.18	0.11	0.0374	0.39	0.696885
Maya	0.78	0.64	0.0442	0.67	0.505123
MbutiPygmy	0.37	0.19	0.1039	0.56	0.572635
Melanesian	0.55	0.39	0.1321	0.44	0.656722
Miao	0.30	0.50	0.0450	-0.95	0.341732
Mongola	0.94	0.59	0.0392	1.73	0.082971
Mozabite	0.47	0.55	0.0468	-0.36	0.719113
Naxi	0.36	0.48	0.0548	-0.48	0.627989
Orcadian	0.51	0.62	0.0459	-0.51	0.609310
Oroqen	0.53	0.62	0.0500	-0.43	0.664401
Palestinian	0.69	0.60	0.0197	0.60	0.546529
Papuan	0.13	0.50	0.1611	-0.91	0.364157
Pathan	0.68	0.82	0.0275	-0.79	0.426714
Pima	0.63	0.73	0.0996	-0.33	0.743225
Russian	0.73	0.56	0.0319	0.96	0.338325
San	0.51	0.19	0.1691	0.79	0.431902
Sardinian	0.79	0.52	0.0342	1.44	0.150713
She	0.68	0.49	0.0498	0.86	0.391621
Sindhi	0.96	0.72	0.0254	1.47	0.142144
Surui	0.95	0.72	0.1718	0.54	0.586772
Tu	0.30	0.53	0.0432	-1.12	0.263329
Tujia	0.28	0.54	0.0411	-1.27	0.204893
Tuscan	0.68	0.62	0.0724	0.23	0.817115
Uygur	1.21	0.66	0.0466	2.57	**0.010253**
Xibo	1.02	0.59	0.0458	2.01	**0.044766**
Yakut	0.59	0.68	0.0401	-0.43	0.664595
Yi	0.41	0.53	0.0413	-0.56	0.577533
Yoruba	0.17	0.10	0.0329	0.39	0.695121
