# Supplementary material for: A Population Genetic Signal of Polygenic Adaptation
Source: PLoS Genet. 2014 Aug 7;10(8):e1004412. doi: 10.1371/journal.pgen.1004412 (PMC4125079; doi:10.1371/journal.pgen.1004412)
Supplement: Table S13 — Conditional analysis at the regional level for the UC dataset. (PDF) [file pgen.1004412.s032.pdf]

	Observed	Expected	Variance	Z	p
Europe	0.30	0.55	0.0144	-2.08	**0.037863**
Middle East	0.43	0.40	0.0127	0.23	0.817361
Central Asia	0.67	0.47	0.0123	1.74	0.081000
East Asia	0.51	0.91	0.0343	-2.12	**0.033839**
Americas	0.43	0.58	0.1149	-0.43	0.667025
Oceania	0.95	0.55	0.1560	1.03	0.305336
Africa	0.33	0.55	0.1229	-0.62	0.532409
