# Supplementary material for: A Population Genetic Signal of Polygenic Adaptation
Source: PLoS Genet. 2014 Aug 7;10(8):e1004412. doi: 10.1371/journal.pgen.1004412 (PMC4125079; doi:10.1371/journal.pgen.1004412)
Supplement: Table S14 — Conditional analysis at the individual population level for the UC dataset. (PDF) [file pgen.1004412.s033.pdf]

	Observed	Expected	Variance	Z	p
Adygei	0.40	0.52	0.0287	-0.75	0.454405
Balochi	0.77	0.65	0.0192	0.80	0.425065
BantuKenya	0.17	0.26	0.0381	-0.44	0.658187
BantuSouthAfrica	0.10	0.31	0.0459	-1.00	0.315538
Basque	0.28	0.36	0.0281	-0.44	0.658278
Bedouin	0.51	0.43	0.0203	0.58	0.561443
BiakaPygmy	0.33	0.37	0.0435	-0.19	0.850748
Brahui	0.69	0.70	0.0212	-0.04	0.966201
Burusho	0.68	0.61	0.0246	0.46	0.644084
Cambodian	0.55	0.47	0.0400	0.37	0.711734
Colombian	0.35	0.50	0.0769	-0.56	0.573665
Dai	0.50	0.46	0.0380	0.24	0.813796
Daur	0.45	0.59	0.0377	-0.69	0.492558
Druze	0.43	0.47	0.0196	-0.31	0.756796
French	0.29	0.38	0.0166	-0.63	0.527907
Han	0.32	0.53	0.0096	-2.13	**0.033117**
Hazara	0.74	0.56	0.0210	1.26	0.207136
Hezhen	0.75	0.47	0.0372	1.44	0.150826
Italian	0.21	0.36	0.0347	-0.79	0.431323
Japanese	0.45	0.51	0.0192	-0.46	0.642099
Kalash	0.26	0.62	0.0563	-1.52	0.127606
Karitiana	0.39	0.41	0.1076	-0.06	0.953921
Lahu	0.63	0.44	0.0600	0.77	0.441513
Makrani	0.72	0.64	0.0194	0.57	0.566336
Mandenka	0.18	0.25	0.0296	-0.37	0.714488
Maya	0.52	0.43	0.0360	0.46	0.646610
MbutiPygmy	0.62	0.40	0.0816	0.78	0.437484
Melanesian	1.14	0.68	0.1018	1.44	0.150504
Miao	0.35	0.49	0.0351	-0.73	0.465745
Mongola	0.69	0.51	0.0305	1.01	0.314222
Mozabite	0.20	0.39	0.0368	-0.98	0.329376
Naxi	0.44	0.51	0.0445	-0.30	0.762696
Orcadian	-0.00	0.39	0.0351	-2.12	**0.034016**
Oroqen	0.57	0.56	0.0373	0.06	0.950027
Palestinian	0.56	0.46	0.0148	0.84	0.399894
Papuan	0.76	0.96	0.1195	-0.55	0.579229
Pathan	0.37	0.68	0.0204	-2.15	**0.031576**
Pima	0.49	0.50	0.0803	-0.04	0.971033
Russian	0.61	0.31	0.0235	1.94	0.052946
San	0.70	0.40	0.1256	0.85	0.393139
Sardinian	0.42	0.29	0.0282	0.76	0.449837
She	0.52	0.46	0.0400	0.31	0.753611
Sindhi	0.86	0.59	0.0199	1.93	0.053454
Surui	0.42	0.53	0.1337	-0.29	0.770331
Tu	0.38	0.48	0.0328	-0.56	0.574730
Tujia	0.51	0.43	0.0324	0.46	0.649040
Tuscan	0.16	0.45	0.0582	-1.17	0.240321
Uygur	0.91	0.53	0.0358	2.00	**0.045954**
Xibo	0.68	0.49	0.0343	0.99	0.323788
Yakut	0.49	0.62	0.0319	-0.74	0.461639
Yi	0.46	0.47	0.0335	-0.05	0.957944
Yoruba	0.23	0.17	0.0250	0.37	0.708225
