# Supplementary material for: A Population Genetic Signal of Polygenic Adaptation
Source: PLoS Genet. 2014 Aug 7;10(8):e1004412. doi: 10.1371/journal.pgen.1004412 (PMC4125079; doi:10.1371/journal.pgen.1004412)
Supplement: Table S15 — Corresponding statistics for all analyses presented in Table 2. (PDF) [file pgen.1004412.s034.pdf]

Phenotype	SUMPC1	SUMPC2	WINPC1	WINPC2	Latitude
Height	-0.22 (0.11)	0.003 (0.93)	-0.12 (0.37)	**0.38 (0.008)**	0.12 (0.34)
Skin Pigmentation	**0.31(0.025)**	0.07 (0.62)	0.27 (0.061)	−0.11 (0.38)	**−0.36 (0.009)**
Body Mass Index	−0.22 (0.15)	0.04 (0.74)	−0.17 (0.29)	0.24 (0.11)	0.21 (0.21)
Type 2 Diabetes	0.10 (0.36)	0.10 (0.42)	0.14 (0.25)	−0.07 (0.56)	−0.20 (0.093)
Crohn's Disease	0.25 (0.066)	**−0.30 (0.033)**	0.01 (0.86)	−0.28 (0.053)	0.08 (0.61)
Ulcerative Colitis	0.17 (0.22)	**−0.29 (0.046)**	0.06 (0.61)	−0.21 (0.15)	−0.11(0.40)
