# Supplementary material for: A Population Genetic Signal of Polygenic Adaptation
Source: PLoS Genet. 2014 Aug 7;10(8):e1004412. doi: 10.1371/journal.pgen.1004412 (PMC4125079; doi:10.1371/journal.pgen.1004412)
Supplement: Table S16 — Corresponding statistics for all analyses presented in Table 2. (PDF) [file pgen.1004412.s035.pdf]

Phenotype	SUMPC1	SUMPC2	WINPC1	WINPC2	Latitude
Height	-0.15 (0.24)	−0.006 (0.89)	-0.13 (0.31)	**0.32 (0.025)**	0.10 (0.42)
Skin Pigmentation	**0.28(0.045)**	0.008 (0.98)	0.24 (0.097)	−0.11 (0.40)	−0.25 (0.087)
Body Mass Index	−0.24 (0.12)	0.07 (0.57)	−0.11 (0.57)	0.25 (0.097)	0.14 (0.47)
Type 2 Diabetes	0.12 (0.30)	0.11 (0.34)	0.09 (0.39)	−0.07 (0.56)	−0.21 (0.08)
Crohn's Disease	0.21 (0.12)	**−0.29 (0.039)**	0.01 (0.86)	−0.22 (0.12)	0.08 (0.63)
Ulcerative Colitis	0.19 (0.17)	−0.23 (0.12)	0.18 (0.65)	−0.15 (0.30)	−0.17(0.20)
